# Cytomegalovirus Downregulates IRE1 to Repress the Unfolded Protein Response

**DOI:** 10.1371/journal.ppat.1003544

**Published:** 2013-08-08

**Authors:** Sebastian Stahl, Julia M. Burkhart, Florian Hinte, Boaz Tirosh, Hermine Mohr, René P. Zahedi, Albert Sickmann, Zsolt Ruzsics, Matthias Budt, Wolfram Brune

**Affiliations:** 1 Heinrich Pette Institute, Leibniz Institute for Experimental Virology, Hamburg, Germany; 2 Division of Viral Infections, Robert Koch Institute, Berlin, Germany; 3 Department of Bioanalytics, ISAS – Leibniz Institute for Analytical Sciences, Dortmund, Germany; 4 Institute for Drug Research, School of Pharmacy, Faculty of Medicine, The Hebrew University, Jerusalem, Israel; 5 Max von Pettenkofer Institute, Ludwig-Maximilians-Universität München, Munich, Germany; 6 Medical Proteome Center (MPC), Ruhr-Universität, Bochum, Germany; 7 DZIF German Center for Infection Research, Munich, Germany; 8 DZIF German Center for Infection Research, Hamburg, Germany; New York University, United States of America

## Abstract

During viral infection, a massive demand for viral glycoproteins can overwhelm the capacity of the protein folding and quality control machinery, leading to an accumulation of unfolded proteins in the endoplasmic reticulum (ER). To restore ER homeostasis, cells initiate the unfolded protein response (UPR) by activating three ER-to-nucleus signaling pathways, of which the inositol-requiring enzyme 1 (IRE1)-dependent pathway is the most conserved. To reduce ER stress, the UPR decreases protein synthesis, increases degradation of unfolded proteins, and upregulates chaperone expression to enhance protein folding. Cytomegaloviruses, as other viral pathogens, modulate the UPR to their own advantage. However, the molecular mechanisms and the viral proteins responsible for UPR modulation remained to be identified. In this study, we investigated the modulation of IRE1 signaling by murine cytomegalovirus (MCMV) and found that IRE1-mediated mRNA splicing and expression of the X-box binding protein 1 (XBP1) is repressed in infected cells. By affinity purification, we identified the viral M50 protein as an IRE1-interacting protein. M50 expression in transfected or MCMV-infected cells induced a substantial downregulation of IRE1 protein levels. The N-terminal conserved region of M50 was found to be required for interaction with and downregulation of IRE1. Moreover, UL50, the human cytomegalovirus (HCMV) homolog of M50, affected IRE1 in the same way. Thus we concluded that IRE1 downregulation represents a previously undescribed viral strategy to curb the UPR.

## Author Summary

Viruses abuse the cell's protein synthesis and folding machinery to produce large amounts of viral proteins. This enforced synthesis overloads the cell's capacity and leads to an accumulation of unfolded proteins in the endoplasmic reticulum (ER) resulting in ER stress, which can compromise cell viability. To restore ER homeostasis, cells initiate the unfolded protein response (UPR) to reduce protein synthesis, increase degradation of unfolded proteins, and upregulate chaperone expression for enhanced protein folding. The most conserved branch of the UPR is the signaling pathway activated by the ER stress sensor IRE1. It upregulates ER-associated degradation (ERAD), thereby antagonizing ER stress. Some of the counter-regulatory mechanisms of the UPR are detrimental for viral replication and are, therefore, moderated by viruses. In this study we identified the first viral IRE1 inhibitor: The murine cytomegalovirus M50 protein, which interacts with IRE1 and induces its degradation. By this means, M50 inhibits IRE1 signaling and prevents ERAD upregulation. Interestingly, the M50 homolog in human cytomegalovirus, UL50, also downregulated IRE1 revealing a previously unknown mechanism of viral host cell manipulation.

## Introduction

During viral replication large amounts of viral proteins must be synthesized, folded, and posttranslationally modified. Folding, maturation and multi-subunit assembly of secreted and transmembrane proteins take place in the endoplasmic reticulum (ER) and require an elaborate system of chaperones, lectins, and carbohydrate-processing enzymes. Whereas correctly folded proteins are transported to the Golgi, misfolded or unfolded proteins are arrested in the ER and diverted for degradation via the ER-associated protein degradation (ERAD) pathway [1]. However, the high levels of viral envelope glycoproteins that are being synthesized particularly during the late phase of the viral life cycle can overwhelm the folding and processing capacity of the ER and cause accumulation of unfolded and misfolded proteins in the ER [2]. In addition, large quantities of secreted and immunomodulatory viral proteins can contribute to ER stress [3]. To reduce protein load and restore ER homeostasis, eukaryotic cells activate various ER-to-nucleus signaling pathways, which are collectively referred to as Unfolded Protein Response (UPR) [4], [5]. The UPR is initiated by three sensor proteins that recognize ER stress: protein kinase R-like ER kinase (PERK), activating transcription factor 6 (ATF6), and inositol-requiring enzyme 1 (IRE1). The ER chaperone BiP (immunoglobulin heavy chain binding protein), also known as glucose-regulated protein 78, is thought to bind these sensors and keep them inactive under normal conditions. However, when unfolded and misfolded proteins accumulate in the ER, BiP dissociates from these sensors to perform its chaperone function. As a consequence, the sensors are activated and initiate UPR signaling. Activation of PERK leads to phosphorylation of the α subunit of eukaryotic translation initiation factor 2 (eIF2α), resulting in global attenuation of protein translation [6], [7]. However, if ER stress persists eIF2α initiates expression of activating transcription factor 4 (ATF4), which induces expression of the proapoptotic transcription factor C/EBP-homologous protein (CHOP, also known as growth arrest and DNA damage-inducible protein 153). CHOP expression promotes apoptosis by downregulating the antiapoptotic protein Bcl-2 [8], [9]. Activated ATF6 translocates to the Golgi where it is cleaved by site 1 and site 2 proteases [10]. The active transcription factor is imported into the nucleus where it induces transcription of chaperone genes [11]. The IRE1 pathway is the most conserved branch of the UPR [12]. Mammalian cells encode two IRE1 isoforms, IRE1α and IRE1β. IRE1α, the most abundant isoform, is expressed in most cells and tissues and is hereafter referred to as IRE1. By contrast, IRE1β (also known as IRE2) is expressed to significant levels only in intestinal epithelial cells [13]. Upon activation, IRE1 dimerizes and transphosphorylates itself. This leads to activation of a site-specific endoribonuclease activity in the cytosolic tail of IRE1, which mediates an unconventional splicing of the X-box binding protein 1 (XBP1) mRNA in the cytosol [14], [15]. The transcription factor XBP1s, which is translated from the spliced *Xbp1* transcript, translocates to the nucleus and induces expression of ERAD enzymes [1], [12]. If ER stress is too severe to overcome and ER homeostasis cannot be restored, IRE1 can also activate c-Jun N-terminal kinase (JNK) to commit damaged cells to apoptosis [16].

Increasing evidence indicates that viruses selectively modulate the UPR to take advantage of the beneficial effects and inhibit those detrimental to viral replication [2]. For instance, hepatitis C virus and other members of the *Flaviviridae* activate beneficial components of the UPR such as BiP in certain cell types to facilitate their replication but trigger ER stress-induced apoptosis in other cells [17]–[20]. Members of the *Herpesviridae* also modulate the UPR to their own advantage. The molecular mechanisms, however, appear to differ from one virus to another [21]. For example the viral glycoprotein gB of herpes simplex virus type 1 (HSV-1) inhibits PERK activation [22]. By contrast, varicella-zoster virus, another alphaherpesvirus, activates the PERK and IRE1 pathways [23]. UPR modulation also takes place in gammaherpesvirus-infected cells. Epstein-Barr virus (EBV) latent membrane protein 1 activates PERK to enhance its own expression [24]. In addition, reactivation of EBV from latent infection is induced by extrinsic ER stress while XBP1 induces EBV lytic gene expression [25]. From these and other examples it has been concluded that UPR regulation plays an important role in viral infection and pathogenesis [2].

Several studies have investigated the ability of human cytomegalovirus (HCMV), a betaherpesvirus, to cope with ER stress and manipulate the UPR to its own benefit. HCMV is a major hazard for immunocompromised individuals such as transplant recipients and the leading infectious cause of birth defects [26]. To enhance viral replication HCMV has adopted several strategies to modulate the UPR. For example, HCMV induces PERK activation, but limits eIF2α phosphorylation. By doing this the virus prevents a global protein synthesis shutoff but allows eIF2α phosphorylation-dependent activation of transcription factor ATF4 [27]. HCMV also uses PERK to induce lipogenesis by activating the cleavage of sterol regulatory element binding protein 1 [28]. In addition, HCMV increases expression of the ER chaperone BiP to facilitate protein folding and virion assembly [29], [30]. Moreover, the viral UL38 protein was shown to prevent ER stress-induced JNK activation and apoptosis [31]. A recent study has revealed that murine cytomegalovirus (MCMV), a related betaherpesvirus, influences the UPR in a similar manner [32]. Particularly, MCMV was shown to activate the PERK–ATF4 pathway and upregulate expression of the ER chaperone BiP. However, in most cases the exact mechanisms by which human and murine cytomegaloviruses modulate the UPR remain undefined.

In the present study, we investigated the influence of MCMV infection on the IRE1 pathway. This pathway has been characterized in yeast as well as in mammalian cells and represents the most evolutionary conserved branch of the UPR [5]. IRE1 mediates an unconventional splicing of *Xbp1*, which in turn triggers expression of ERAD proteins [33]. We discovered an interaction between IRE1 and the viral protein M50. The viral M50 was previously characterized as a type II transmembrane (TM) protein that associates with the viral M53 protein. M50 and M53 are essential components of a complex that dissolves the nuclear lamina [34]. Proteins homologous to M50 are found in all herpesviruses studied thus far, and these proteins are involved in nuclear egress of viral capsids. Moreover, M50 and its homologs are essential for lytic replication of beta- and gammaherpesviruses [35], [36]. We show that M50 expression induces a robust downmodulation of IRE1 levels in transfection and infection experiments suggesting that M50 induces IRE1 degradation. The N-terminal conserved region of M50 proved to be required for IRE1 binding and degradation. We further showed that UL50, the HCMV homolog of M50, has a similar function. We propose that inhibition of IRE1 signaling by removal of the sensor IRE1 represents a previously unrecognized viral strategy to curb the UPR.

## Results

### MCMV inhibits the IRE1-dependent UPR branch

**Figure 1:**
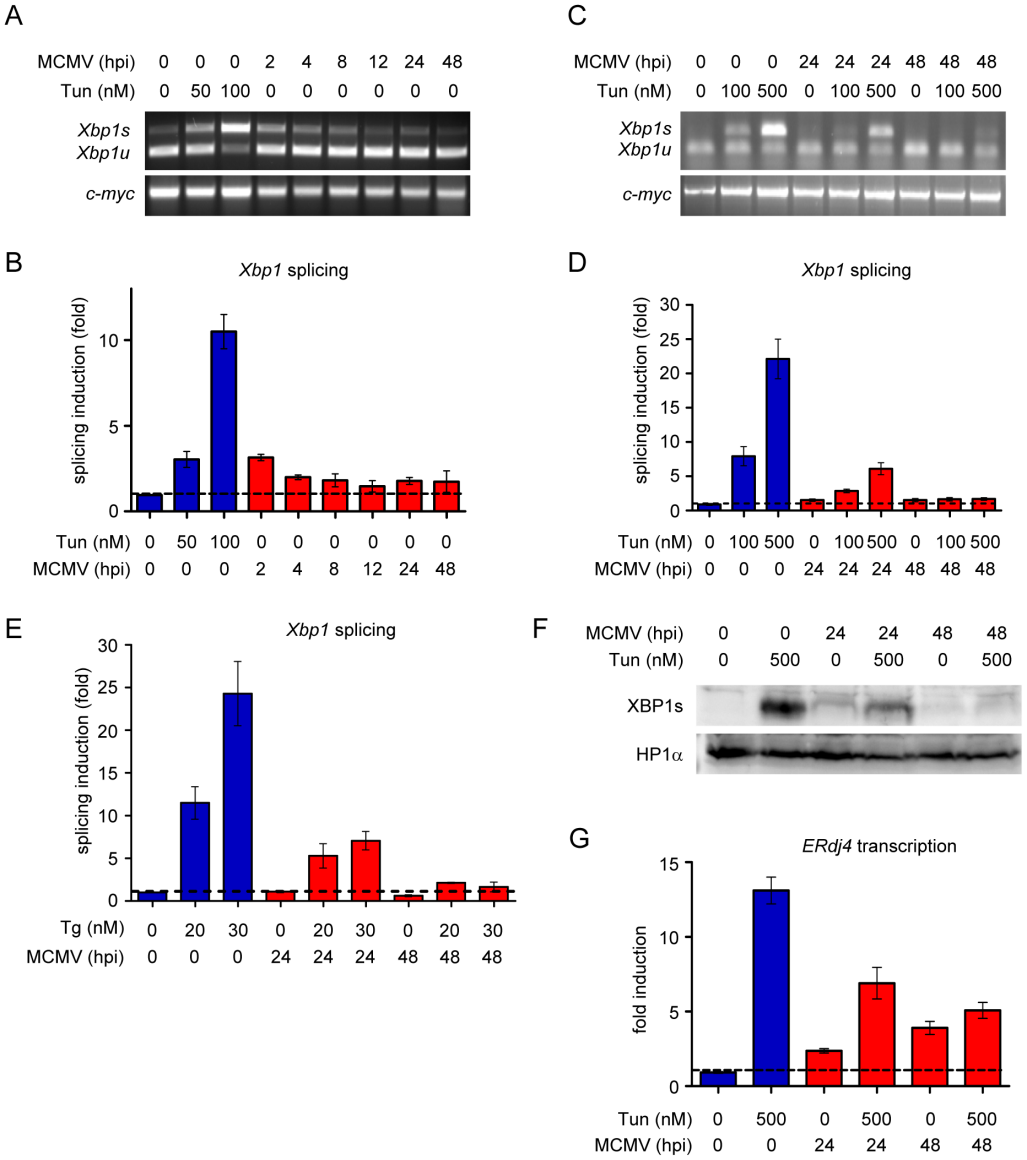
MCMV modulates *Xbp1* splicing. (A) NIH-3T3 cells were infected with MCMV at an MOI of 5 or treated with tunicamycin (Tun) for 4 h. *Xbp1* mRNA transcripts were amplified by RT-PCR, digested with PstI, and separated on an ethidium bromide-stained agarose gel. The spliced transcript, *Xbp1s*, lacks the PstI site and migrates slower than the digested unspliced transcript, *Xbp1u*. (B) NIH-3T3 cells were treated as described for panel A. *Xbp1s* and *Xbp1u* transcripts were quantified by real-time RT-PCR. Changes in the *Xbp1s*/*Xbp1u* ratio relative to untreated cells are plotted as bar diagram showing means ±SEM of four replicates. (C) NIH-3T3 cells were infected as above and treated in addition with Tun for the last 4 h before harvest. *Xbp1* transcripts were analyzed as in A. (D) NIH-3T3 cells were treated as in C, and *Xbp1* transcripts were quantified as in B. (E) NIH-3T3 cells were MCMV-infected and treated with thapsigargin (Tg) for the last 4 h before harvest. *Xbp1* transcripts were quantified as in B. (F) NIH-3T3 cells were MCMV-infected and treated with Tun for the last 4 h before harvest. Nuclear protein extracts were analyzed by immunoblot for the presence of XBP1s protein. Heterochromatin protein 1α (HP1α) was used as a loading control. (G) NIH-3T3 cells were infected with MCMV at an MOI of 5 and treated with Tun for 4 h. *ERdj4* transcripts were quantified by real-time RT-PCR. Results are shown as fold induction relative to untreated cells (means ±SEM of four replicates).

As it has been shown that cytomegaloviruses inhibit the IRE1-dependent UPR pathway by an unknown mechanism [27], [32], we wanted to investigate how MCMV modulates this pathway. First, we measured *Xbp1* mRNA splicing during MCMV infection by semiquantitative RT-PCR and real-time RT-PCR. Since the 26 nt intron, which is spliced out by IRE1, contains a PstI restriction site [14], [15], only the unspliced RT-PCR product is cleaved by PstI. MCMV infection of NIH-3T3 fibroblasts induced a slight and transient increase in *Xbp1* splicing similar to the one induced by treatment with a very low dose of tunicamycin (Tun), an established ER stress inducer (Fig. 1A and B). The ratio of spliced to unspliced transcripts returned almost to baseline levels around 8 hours postinfection (hpi) and remained constant until 48 hpi. To test whether MCMV actively suppresses *Xbp1* splicing, we treated MCMV-infected fibroblasts with Tun and measured *Xbp1* splicing. As shown in Figure 1C and D, Tun-induced *Xbp1* splicing was strongly reduced at 24 hpi and almost completely blocked at 48 hpi. A similar inhibition of *Xbp1* splicing was observed when infected cells were treated with the ER stress inducer thapsigargin (Fig. 1E). We also determined the protein levels of transcription factor XBP1s by immunoblot analysis. Consistent with the RT-PCR results, Tun-induced XBP1s protein expression was inhibited at 24 and almost completely blocked at 48 hpi (Fig. 1F). Moreover, ER stress-induced transcription of the XBP1s target gene *ERdj4*, which encodes an ERAD protein [37], was also inhibited (Fig. 1G), further confirming the conclusion that MCMV actively inhibits the IRE1 pathway at late times postinfection.

### MCMV M50 interacts with IRE1

**Figure 2:**
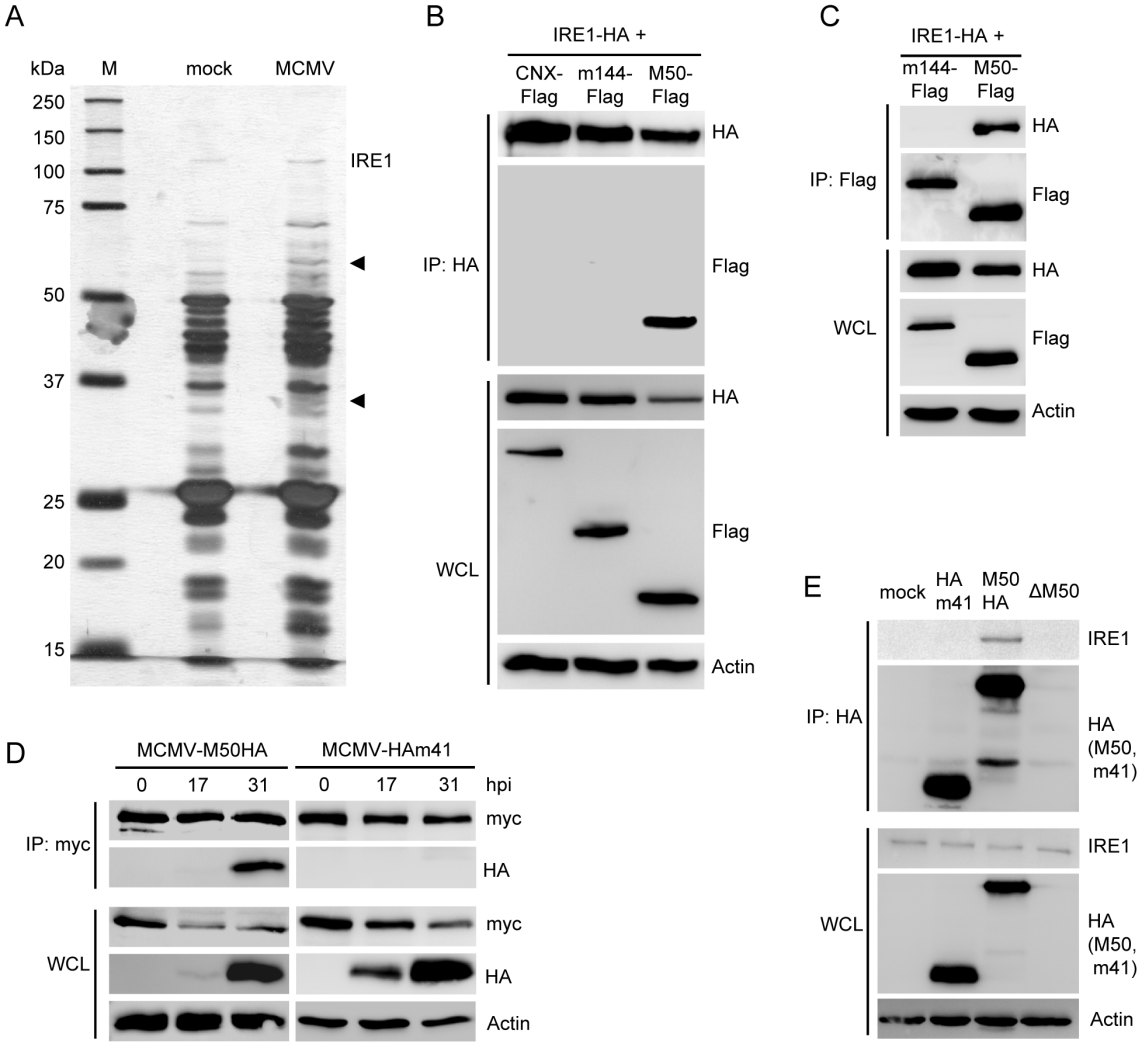
MCMV M50 interacts with IRE1. (A) NIH-3T3 cells stably expressing TEV-HA-tagged IRE1 were mock infected or infected with MCMV at an MOI of 5. Whole cell lysates (WCL) were applied to an anti-HA sepharose matrix. IRE1 and interacting proteins were eluted by TEV protease digestion, separated by SDS-PAGE, and silver stained. Specific bands (arrow heads) were excised and analyzed by protein mass spectrometry. (B) 293A cells were cotransfected with expression plasmids for IRE1-HA and Flag-tagged M50, m144, or Calnexin (CNX), respectively. IRE1 was subjected to immunoprecipitation (IP) with an anti-HA antibody. IRE1 and coexpressed proteins were detected by immunoblot in IP samples and WCL using anti-HA and anti-Flag antibodies, respectively. (C) 293A cells were cotransfected with expression plasmids for IRE1-HA and Flag-tagged M50 or m144, respectively. M50 and m144 were precipitated with an anti-Flag antibody. IRE1 and coexpressed proteins were detected in IP samples and WCL as described above. (D) 10.1 fibroblasts transduced with a retroviral vector expressing myc-tagged IRE1 were infected with an MCMV expressing HA-tagged M50 or m41 at an MOI of 4. At the indicated time points IRE1 was immunoprecipitated with an anti-myc antibody, and HA-tagged proteins were detected by immunoblot. (E) NIH-3T3 cells were infected with the same viruses as in D. After 24 h, M50 and m41 proteins were immunoprecipitated with an anti-HA antibody. IRE1 was detected in IP samples and WCL using an IRE1-specific antibody.

As activated IRE1 is the only enzyme mediating *Xbp1* mRNA splicing, we hypothesized that MCMV might express a protein that interacts with IRE1. To identify IRE1 interaction partners, we stably transfected NIH-3T3 cells with a plasmid encoding IRE1 with a C-terminal tobacco etch virus (TEV) protease cleavage site and an HA epitope tag. IRE1-TEV-HA-expressing cells were infected with MCMV, and protein lysates were loaded onto an anti-HA affinity matrix. After washing, IRE1 was released from the matrix by TEV protease digestion. Eluted proteins were separated by gel electrophoresis and silver stained (Fig. 2A). Bands not present in the control lane (uninfected cells) were excised and analyzed by protein mass spectrometry. In an approx. 32 kDa band two MCMV proteins were identified: M50 and M85. M50 is a type II transmembrane (TM) protein with a C-terminal TM anchor. It is found in the ER membrane and the nuclear envelope and is known to play a crucial role in nuclear egress of viral capsids [34], [38]–[40]. M85 is the MCMV minor capsid protein [41] and is not known to be associated with ER membranes. To confirm or dismiss the two MCMV proteins as specific interaction partners of IRE1, HEK 293 cells were transfected with expression plasmids encoding HA-tagged IRE1 and Flag-tagged MCMV proteins. Flag-tagged M50 coprecipitated with IRE1-HA, and IRE1-HA coprecipitated with M50-Flag (Fig. 2B and C). IRE1 did not interact with Flag-tagged m144 or Calnexin (CNX), two ER-localized control proteins. IRE1 also did not interact with Flag-tagged M85 in co-immunoprecipitation experiments (data not shown). Therefore, M85 was not further investigated as a modulator of the IRE1 signaling pathway.

Next we tested whether IRE1 interacts with M50 during MCMV infection. As endogenous IRE1 is expressed at low levels and is difficult to analyze, we used cells expressing epitope-tagged IRE1 from a retroviral vector – a procedure used in several previous studies [42], [43]. 10.1 fibroblasts stably expressing myc-tagged IRE1 were infected with MCMV mutants expressing HA-tagged M50 (MCMV-M50HA) or HA-tagged m41 (MCMV-HAm41). Cell lysates were harvested 17 and 31 hpi and subjected to immunoprecipitation and immunoblot analyses. Fig. 2D shows that M50 coprecipitated with IRE1, consistent with the affinity purification experiment (Fig. 2A), but m41, an unrelated MCMV type 2 TM protein [44], did not. Likewise, HA-tagged M50, but not m41, interacted with endogenous IRE1 in MCMV-infected NIH-3T3 cells (Fig. 2E).

### M50 colocalizes with IRE1

**Figure 3:**
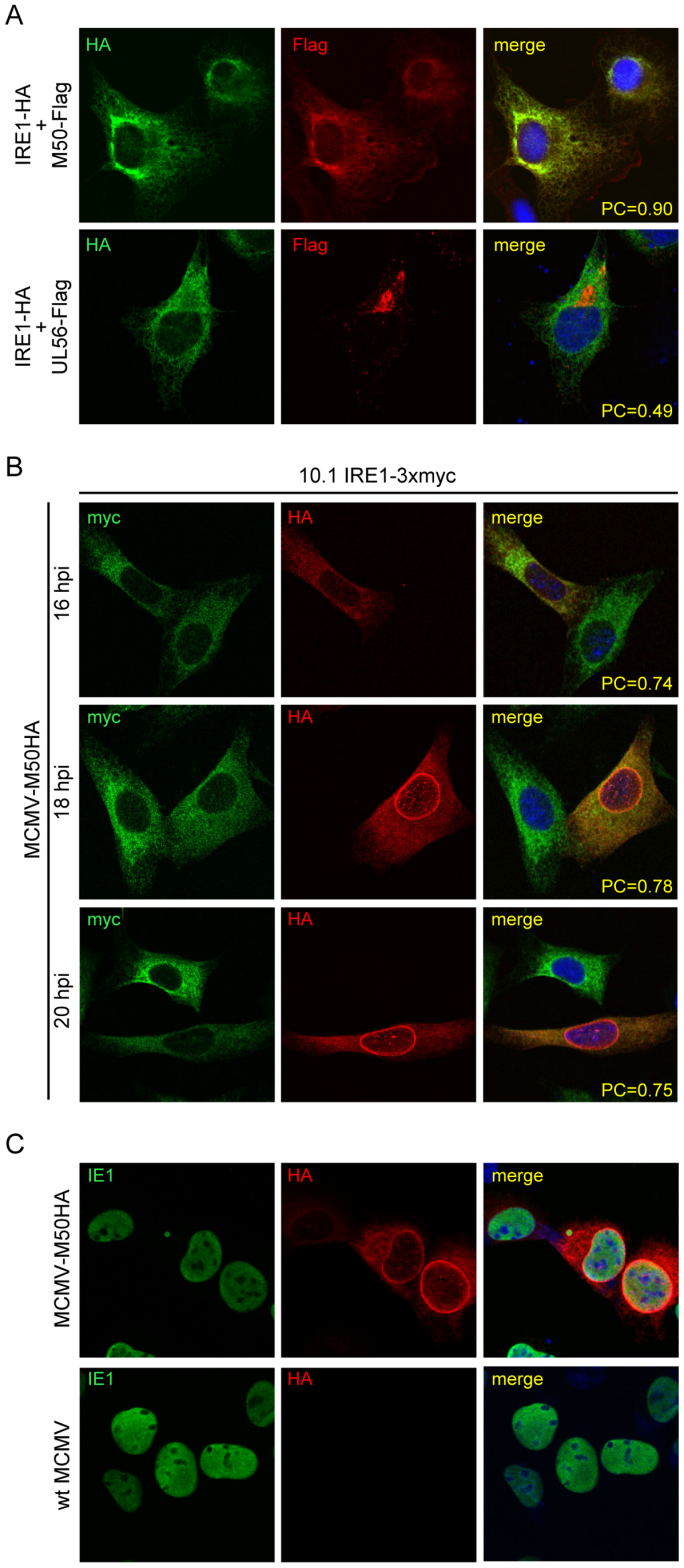
Intracellular localization of IRE1 and M50. (A) NIH-3T3 cells were cotransfected with expression plasmids for IRE1-HA and Flag-tagged M50 or UL56 respectively. 24 h post transfection, cells were fixed and subjected to immunofluorescence staining using HA- and Flag-specific antibodies. Cell nuclei were stained with Draq5. The Pearson correlation coefficient (PC) was determined for transfected cells. (B) 10.1 cells stably expressing IRE1-3xmyc were infected with MCMV-M50HA. At 16, 18, and 20 hpi cells were fixed and subjected to immunofluorescence staining using myc- and HA-specific antibodies. (C) 10.1 cells were infected with wt MCMV or MCMV-M50HA. Cells were fixed 20 hpi and stained with the same anti-HA antibody as in B and an antibody against the viral IE1 protein.

Next we analyzed the subcellular localization of IRE1 and M50 by immunofluorescence (IF). To do this, we cotransfected cells with expression plasmids for IRE1 and M50 or UL56, an unrelated type 2 TM protein of Herpes Simplex Virus type 1 [45]. As shown in Figure 3A, IRE1 and M50 colocalized in transfected NIH-3T3 cells, but IRE1 and UL56 did not.

We also tested whether IRE1 and M50 colocalize in MCMV-infected fibroblasts. As M50 is a late protein, infected cells had to be fixed and stained at late time points, but not too late in order to avoid cell rounding and detachment as a result of the MCMV-induced cytopathic effect. Moreover, M50 is known to change its localization during MCMV infection: it first localizes to the ER, but is subsequently redistributed to the nuclear rim as a consequence of its interaction with the nuclear MCMV protein M53 [34], [40]. When we infected 10.1 fibroblasts expressing myc-tagged IRE1 with MCMV-M50HA, we saw that a substantial portion of M50 retained a cytoplasmic distribution despite an obvious accumulation at the nuclear rim, and this portion of M50 colocalized with IRE1 (Fig. 3B). We also noticed that IRE1 levels appeared to be reduced at late times in MCMV-infected cells compared to neighboring uninfected cells (Fig. 3B, 20 hpi).

To rule out the possibility that the detection of HA-tagged M50 in the cytoplasm resulted from an unspecific binding of the anti-HA antibody to MCMV-infected cells, fibroblasts were infected with wt MCMV or MCMV-M50HA and subjected to IF analysis. Using the same anti-HA antibody and the same staining conditions, a cytoplasmic staining was detected only in MCMV-M50HA-, but not in wt MCMV-infected cells (Fig. 3C).

### M50 expression reduces IRE1 protein levels

**Figure 4:**
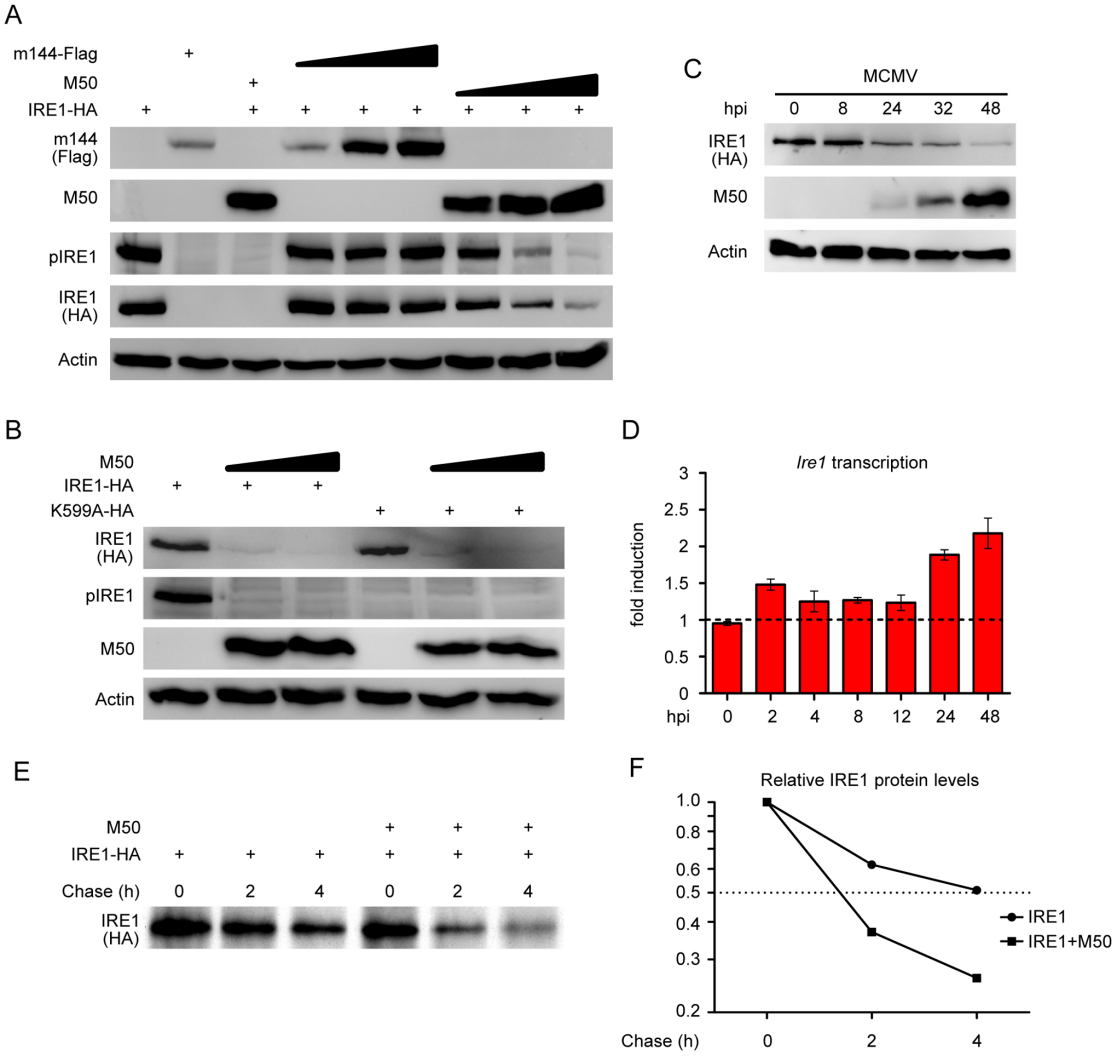
M50 expression reduces IRE1 protein levels. (A) NIH-3T3 cells were cotransfected with plasmids encoding IRE1-HA (1 µg) and Flag-tagged M50 or m144 (0.5, 1, or 2 µg). After 24 h, cell lysates were analyzed by immunoblot using protein- or tag-specific antibodies. (B) NIH-3T3 cells were cotransfected with plasmids encoding M50 (2 or 3 µg) and wildtype IRE1 or the K599A kinase-dead IRE1 mutant (1 µg). Cells were analyzed by immunoblot as described above. (C) 10.1 cells transduced with an IRE1-HA-expressing retroviral vector were infected with MCMV at an MOI of 5. Cells were harvested at the indicated time points, and IRE1, M50, and actin levels were determined by immunoblot using protein- or tag-specific antibodies. (D) NIH-3T3 fibroblasts were infected with MCMV at an MOI of 5. RNA was isolated at the indicated time points, and *Ire1* transcripts were quantified by real-time RT-PCR. Means ±SEM of three replicates are shown relative to uninfected cells. (E) 293T cells were cotransfected with expression plasmids for IRE1-HA and M50. After pulse-chase labeling with [^35^S]methionine, IRE1 was immunoprecipitated with an anti-HA antibody and analyzed by autoradiography. (F) Signals in blot E were quantified by densitometry relative to the 0 h chase value.

Next we investigated whether M50 inhibits IRE1 phosphorylation, which is required for activation of its endoribonuclease activity. To do this, we transfected NIH-3T3 cells with an IRE1 expression plasmid and cotransfected increasing amounts of an M50 or an m144 expression plasmid. Overexpression of IRE1 is known to cause its activation by autotransphosphorylation [46]. As shown in figure 4A, the levels of phosphorylated IRE1 decreased with increasing M50 expression but not with increasing expression of the m144 control protein. Moreover, total IRE1 levels were also decreased, indicating that M50 reduces IRE1 levels rather than just inhibiting its activation. Nevertheless, IRE1 phosphorylation might be required for its downregulation. To test this hypothesis, NIH-3T3 cells were cotransfected with expression plasmids for M50 and either wildtype (wt) IRE1 or a kinase-inactive mutant (K599A). As expected, overexpressed IRE1 K599A was not phosphorylated. However, it was downregulated by M50 just like wt IRE1 (Fig. 4B). Thus we concluded that IRE1 downregulation is independent of its phosphorylation.

As M50 also interacts with the viral M53 protein at the nuclear envelope, we tested whether M53 expression affects the M50-induced IRE1 downregulation. NIH-3T3 cells were cotransfected with plasmids encoding IRE1, M50, and M53. As shown in figure S1, M50 expression induced IRE1 downregulation also in the presence of M53. However, downregulation was reduced when larger amounts of M53 expression plasmid were cotransfected, suggesting that M53 and IRE1 compete for binding to M50.

To check whether IRE1 was also downregulated during MCMV infection, IRE1 levels were determined in an infection time course experiment. Figure 4C shows that IRE1 levels decreased during the course of infection as M50 levels increased. The observed IRE1 downregulation is consistent with the inhibited *Xbp1* splicing in normal fibroblasts, which express only endogenous IRE1 (Fig. 1)

Next we investigated whether IRE1 downregulation occurred at the transcriptional level. RNA was isolated from MCMV-infected cells and *Ire1* transcripts were quantified by real-time RT-PCR. The results showed that *Ire1* transcripts did not decrease but rather increased slightly during the course of MCMV infection (Fig. 4D), indicating that IRE1 downregulation occurred at the posttranscriptional level.

We then tested whether M50 induces IRE1 degradation. To do this, HEK 293 cells were cotransfected with M50 and IRE1 expression plasmids, and IRE1 stability was determined by pulse-chase analysis. Indeed, IRE1 stability was reduced significantly when M50 was coexpressed (Fig. 4E and F), strongly suggesting that M50 induces IRE1 degradation. To test whether ubiquitylation was necessary for IRE1 degradation, IRE1 and M50 were coexpressed in ts20 cells, which have a temperature sensitive E1 ubiquitin-activating enzyme [47]. In these cells, M50 expression reduced IRE1 levels at both the permissive and restrictive temperatures (Fig. S2A), suggesting that ubiquitin conjugation was not required. The IRE1 downregulation seen in immunoblot experiments was also not inhibited by proteasome inhibitors MG132 or lactacystin (Fig. S2B). We also investigated whether IRE1 degradation could be inhibited by lysosomal protease inhibitors (PI) or NH_4_Cl, an inhibitor of lysosome acidification. Neither NH_4_Cl nor a PI cocktail inhibited IRE1 downregulation by M50 (Fig. S2C). Collectively these data suggested that IRE1 is degraded neither by the proteasome nor in lysosomes but rather cleaved by another cellular protease. It is also conceivable that lysosomal proteases that are not inhibited by these drugs are responsible for IRE1 degradation.

### The M50 conserved region is required for IRE1 downregulation

**Figure 5:**
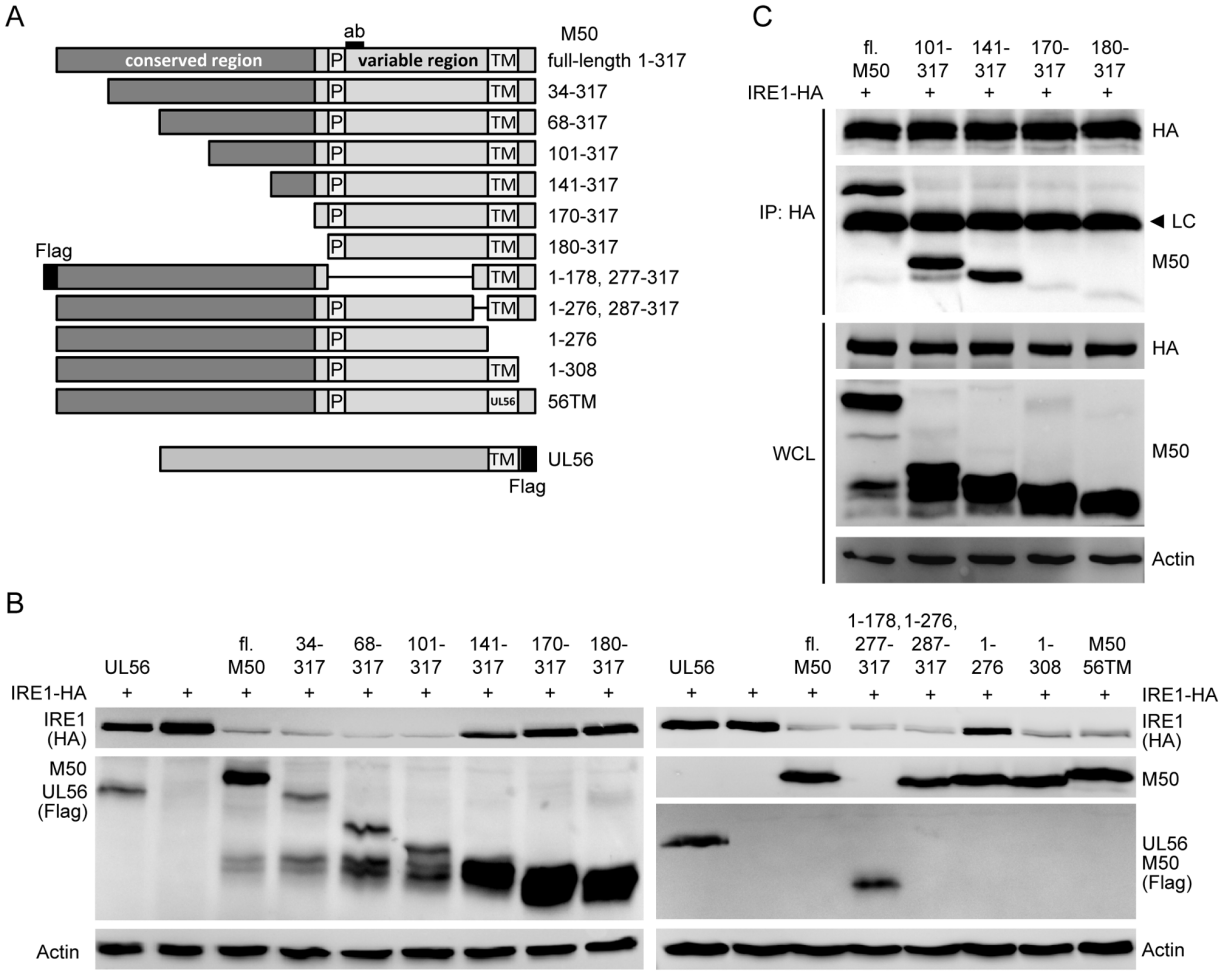
Identification of the region required for IRE1 binding and degradation. (A) Schematic representation of the mutant M50 proteins used in the following experiments. Proline-rich (P) sequence, transmembrane (TM) domain, and the peptide recognized by the M50-specific antibody (ab) are indicated. Numbers on the right indicate amino acid positions. The HSV-1 UL56 protein was used as an unrelated control protein. 56TM is an M50 mutant containing the TM domain of HSV-1 UL56. (B) NIH-3T3 cells were cotransfected with plasmids coding for IRE1-HA (1 µg) and the proteins shown in panel A (2 µg). After 24 h, IRE1 levels were analyzed by immunoblot using an anti-HA antibody. M50 mutants and UL56 were detected with M50- and Flag-specific antibodies. (C) 293A cells were cotransfected with expression plasmids for IRE1-HA and full-length (fl.) or mutant M50. IRE1 was immunoprecipitated (IP) with an anti-HA antibody, and coprecipitating M50 proteins were detected by immunoblot using an M50-specific antibody. The same proteins were detected in whole cell lysates (WCL). LC, antibody light chain.

M50 consists of an N-terminal conserved region, a variable region, a TM domain, and a short C-terminal tail [39]. The N-terminal region is conserved among the herpesviruses, particularly those of the same subfamily [48]. To determine which parts of M50 are required for IRE1 downregulation, a number of N- and C-terminal truncation mutants and mutants with internal deletions were constructed (Fig. 5A). These mutants were tested for their ability to downregulate IRE1 levels and interact with IRE1. In cotransfection experiments, M50 mutants lacking the entire conserved region were unable to downregulate IRE1, whereas mutants lacking only a part of the conserved or the variable region downregulated IRE1 (Fig. 5B). The 141–317 mutant repeatedly displayed an intermediate phenotype, i.e. a moderate downregulation of IRE1. Truncated M50 proteins lacking up to 140 aa from the N-terminus coprecipitated with IRE1, but mutants lacking the entire conserved region did not (Fig. 5C). The M50 1–276 mutant, which lacks the TM domain, was also incapable of downregulating IRE1 (Fig. 5B) but coprecipitated with IRE1 (Fig. S3A) and colocalized, at least partially, with IRE1 in transfected cells (Fig. S3B). However, when the M50 TM domain was substituted by the TM domain of an unrelated type 2 TM protein, HSV-1 UL56, IRE1 downregulation was restored, suggesting that the M50 protein needs a TM anchor for IRE1 downregulation but not for interaction with IRE1.

### M50 downregulates IRE1 during MCMV infection

**Figure 6:**
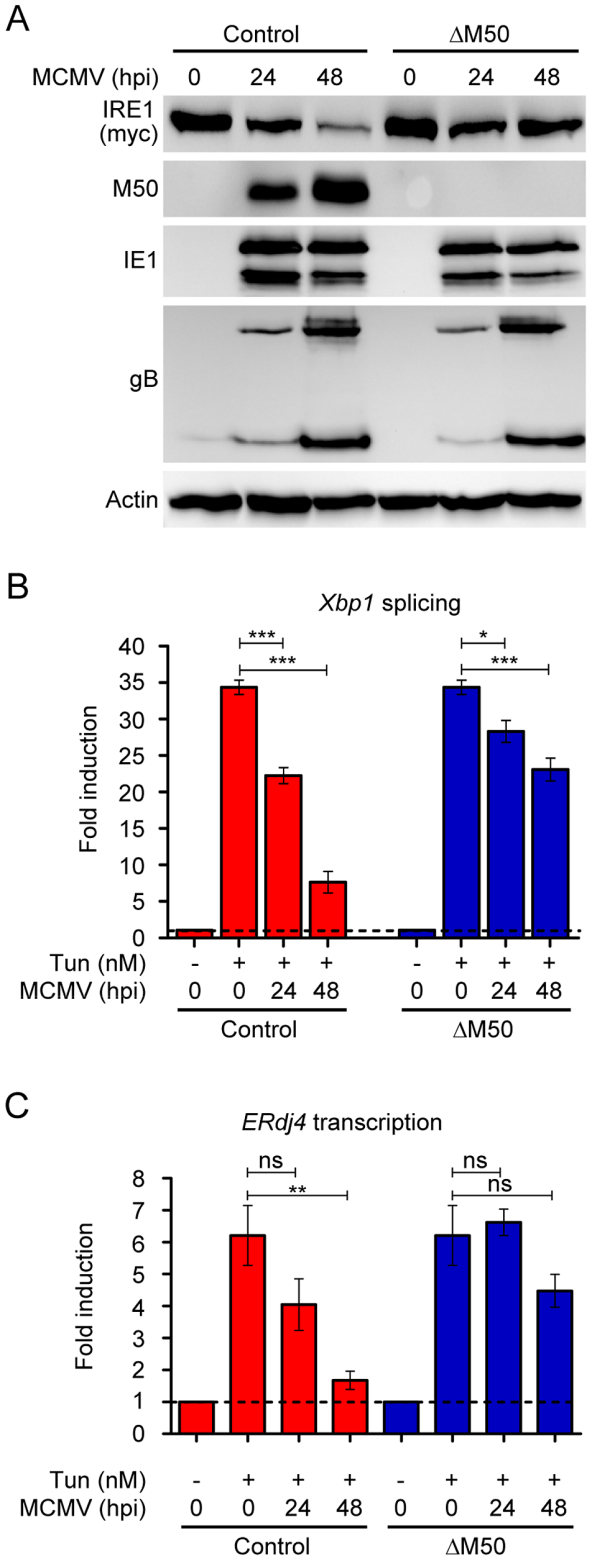
M50 is required for IRE1 downregulation and inhibition of *Xbp1* splicing during MCMV infection. (A) 10.1 fibroblasts stably expressing myc-tagged IRE1 were infected with an MCMV M50 deletion mutant (ΔM50) or the parental control virus at an MOI of 3. Cells were harvested at 0, 24, and 48 hpi. IRE1 and M50 expression was determined by immunoblot. The viral immediate-early 1 (IE1), the viral late protein M55/gB, and cellular β-actin were used as infection and loading controls, respectively. (B) Normal 10.1 fibroblasts were infected as described above and treated for 4 h with Tun. Spliced and unspliced *Xbp1* transcripts were quantified by real-time RT-PCR. Changes in the spliced/unspliced ratio relative to untreated cells are plotted as bar diagram showing means ±SEM of four replicates. (C) *ERdj4* transcripts were quantified in the same cell by real-time RT-PCR. Results are shown as fold induction relative to untreated cells (means ±SEM of three replicates). Significance was determined using the Student's *t*-test. *, p<0.5; **, p<0.01; ***, p<0.001; ns, not significant.

We wanted to test whether M50 is responsible for the IRE1 downregulation observed in MCMV-infected cells (Fig. 4C). This could be done with an MCMV M50 deletion mutant or a virus mutant expressing an M50 protein lacking the conserved region. Unfortunately, M50 is essential for MCMV replication as it mediates, together with M53, nuclear egress of viral capsids [34]. The conserved region of M50, which mediates interaction with IRE1 (Fig. 5), is also required for its interaction with M53 [38], [39]. Until recently, all attempts to generate M50 trans-complementing cell lines for the propagation of an M50-deficient MCMV had failed because stable M50 expression was not tolerated by cells [34]. This obstacle was recently overcome with an MCMV-inducible expression system based on an episomal replicating plasmid containing the MCMV origin of lytic replication and the M50 gene [49]. In NIH-3T3 cells stably carrying this plasmid M50 expression was silenced. Upon MCMV infection, however, the vector was replicated and M50 expression was strongly induced. An MCMV mutant lacking M50 (MCMVΔM50) could be propagated on these trans-complementing cells [49] and used for further experiments. When we infected 10.1 fibroblasts stably expressing myc-tagged IRE1 with MCMVΔM50 or the parental control virus, we observed a strong downregulation of IRE1 levels by the parental MCMV, but only a slight reduction by the MCMVΔM50 virus (Fig. 6A). MCMV infection caused a modest reduction of *Ire1* transcripts in these cells (Fig. S4). This reduction was seen for both viruses, indicating that M50 is not responsible for this effect. However, it is possible that the slightly reduced IRE1 protein levels observed in MCMVΔM50-infected cells (Fig. 6A) were caused by reduced *Ire1* transcription. In NIH-3T3 fibroblasts (expressing only endogenous IRE1), splicing of *Xbp1* transcripts and transcription of *ERdj4* was strongly inhibited upon infection with the MCMV control virus, but only moderately diminished upon infection with MCMVΔM50 (Fig. 6B and C). These results showed that M50 is primarily responsible for inhibition of the IRE1-XBP1 pathway during MCMV infection. However, the moderate reduction in *Xbp1* mRNA splicing and *ERdj4* transcription in MCMVΔM50-infected cells suggest that additional mechanisms contribute to the inhibition of the IRE1-XBP1 signaling pathway.

### HCMV UL50 interacts with IRE1 and mediates IRE1 downregulation

**Figure 7:**
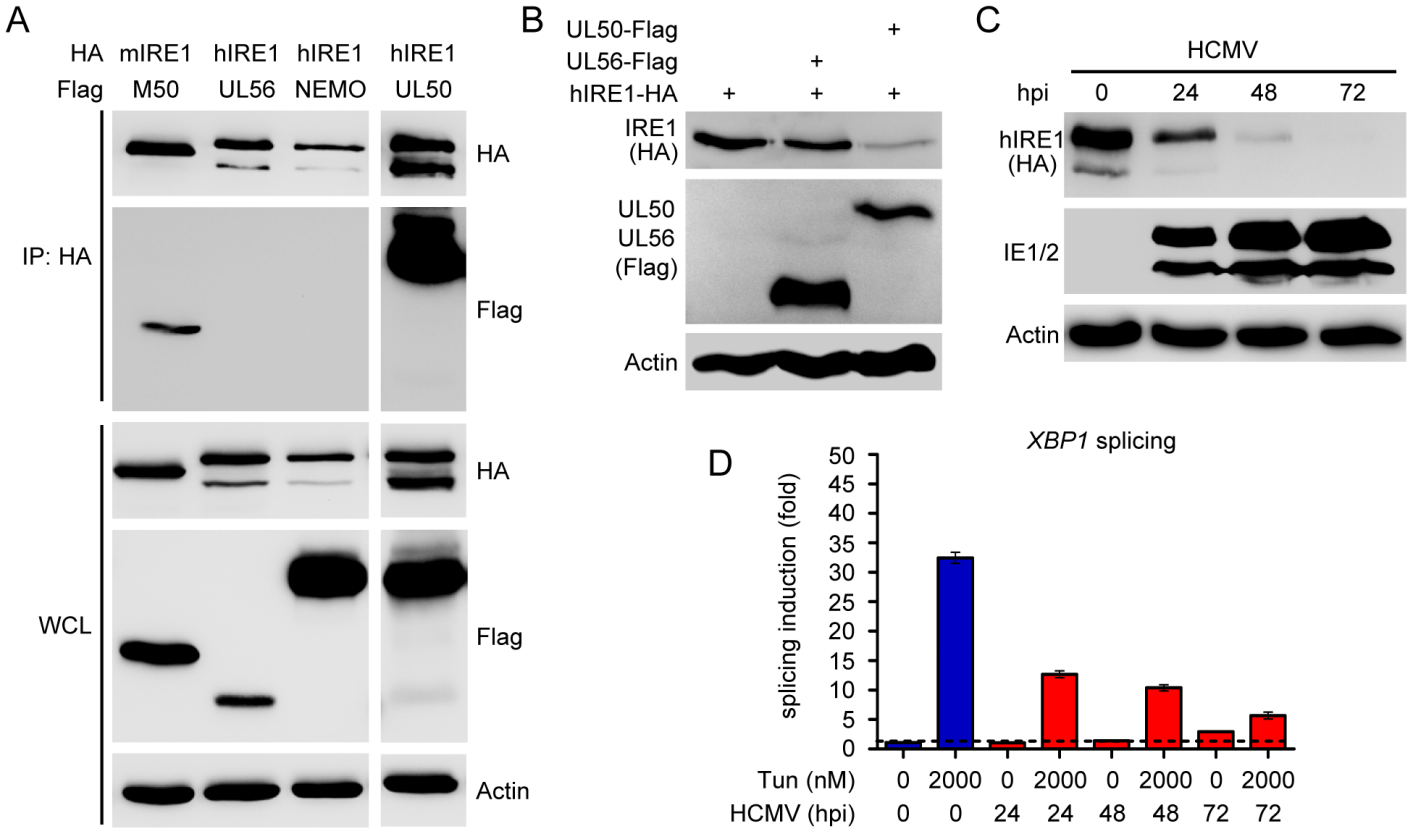
Modulation of the IRE1-XBP1 pathway by HCMV UL50. (A) 293A cells were cotransfected with plasmids expressing HA-tagged murine or human IRE1 and Flag-tagged M50, UL56, NEMO, or UL50 respectively. Cell lysates were harvested 24 h after transfection. IRE1 was immunoprecipitated (IP) with an anti-HA antibody. IRE1 and coexpressed proteins were detected by immunoblot in IP samples and whole cell lysates (WCL) using anti-HA and anti-Flag antibodies, respectively. (B) HFF cells were cotransfected with plasmids encoding IRE1-HA (1 µg) and Flag-tagged HCMV UL50 or HSV-1 UL56 (2 µg). After 24 h, IRE1 levels were determined by immunoblot using an anti-HA antibody. UL50 and UL56 were detected with an anti-Flag antibody. (C) MRC-5 cells transduced with a retroviral vector expressing HA-tagged human IRE1 were infected with HCMV at an MOI of 3. At the indicated time points cells were harvested, and IRE1 levels were determined by immunoblot. HCMV IE1 and IE2 and β-actin were detected as infection and loading controls, respectively. (D) MRC-5 cells were infected with HCMV at an MOI of 3 and treated with Tun for the last 4 h before harvest. Spliced and unspliced *XBP1* transcripts were quantified by real-time RT-PCR. Changes in the spliced/unspliced ratio are shown relative to untreated cells (means ±SEM of three replicates).

M50 is a protein conserved among the *Herpesviridae* family, and the functional conservation was reported to be particularly strong among members of the same subfamily [48]. Hence we tested whether UL50, the HCMV homolog of M50, has a similar function. Indeed, UL50 coimmunoprecipitated with IRE1 like M50 did (Fig. 7A), and UL50 expression downregulated IRE1 levels in transfected cells (Fig. 7B). Moreover, IRE1 levels in HCMV-infected fibroblasts decreased over time (Fig. 7C), and this decrease correlated with a suppression of *Xbp1* splicing following Tun treatment (Fig. 7D). Therefore we concluded that the novel function of M50 described in this report is not unique for MCMV but conserved in the related human pathogen, HCMV.

## Discussion

**Figure 8:**
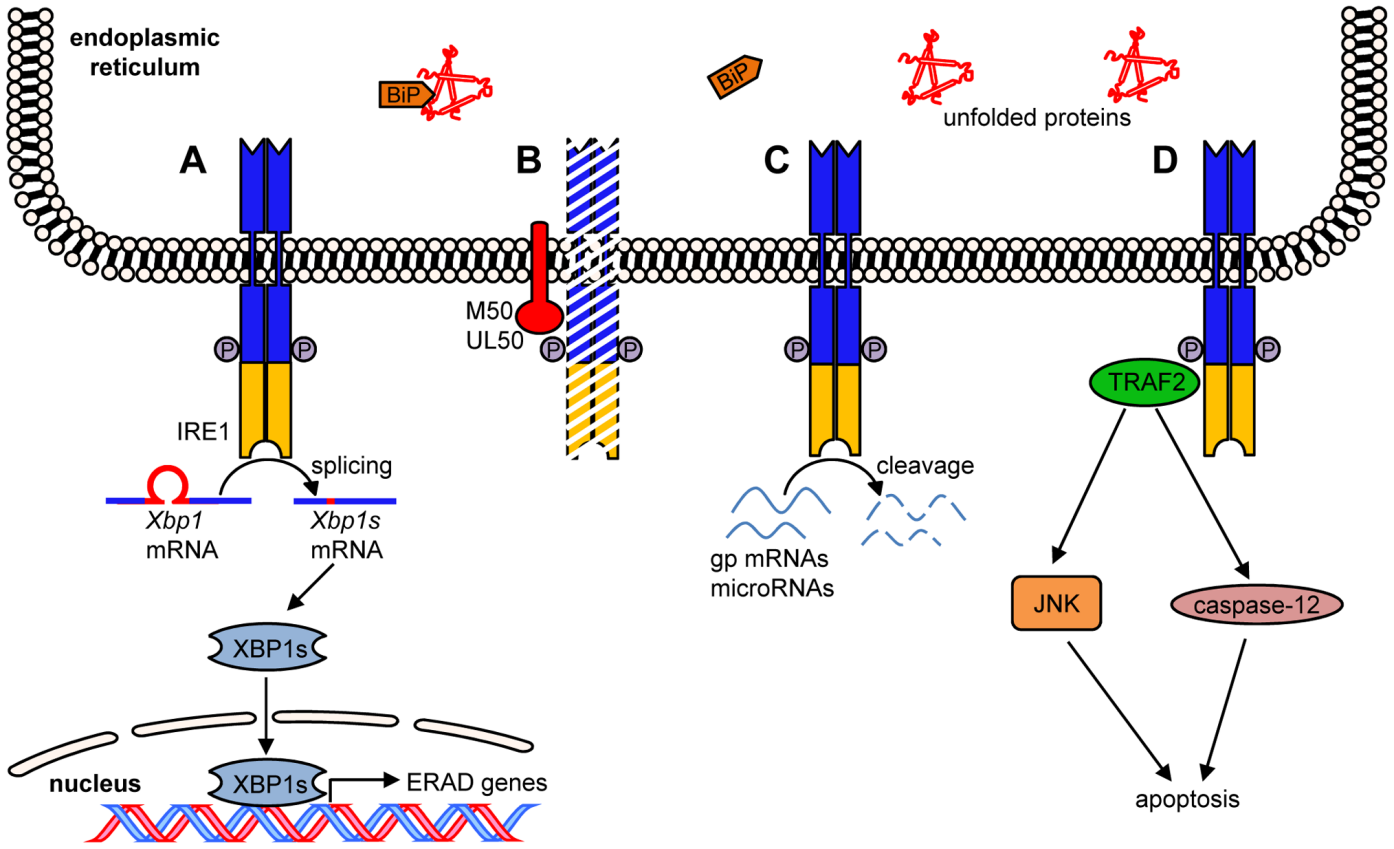
IRE1 functions and inhibition by M50 and UL50. Accumulation of unfolded proteins in the ER leads to recruitment of chaperones such as BiP and activation of ER stress sensors such as IRE1. (A) IRE1 dimerizes, autophosphorylates itself, and activates an endoribonuclease activity, which mediates *Xbp1* mRNA splicing. The XBP1s protein activates transcription of ERAD genes such as *ERdj4*. (B) MCMV M50 and HCMV UL50 interact with IRE1 and induce IRE1 degradation, thereby inhibiting the IRE1-XBP1 pathway shown in A. (C) Activated IRE1 can also cleave certain glycoprotein (gp)-encoding mRNAs and microRNAs. (D) Recruitment of TRAF2 by activated IRE1 can lead to JNK or caspase-12 activation and subsequent induction of apoptosis.

In this study we showed that MCMV and HCMV repress IRE1-mediated ER-to-nucleus signaling, the most conserved branch of the UPR (Fig. 8A). The viral proteins M50 and UL50, respectively, interact with IRE1 and downregulate IRE1 levels in transfected or infected cells (Fig. 8B). Thereby, IRE1-mediated *Xbp1* mRNA splicing, synthesis of transcription factor XBP1s, and expression of XBP1s target genes are inhibited. These results are consistent with two previous studies, which have reported an inhibition of EDEM (an XBP1s target gene) expression by HCMV [27] and a block to *Xbp1* mRNA splicing by MCMV [32], respectively. In these studies, the underlying mechanism of these effects and the viral proteins involved were not investigated. However, other previous studies have shown that HCMV upregulates the ER chaperone BiP through increased transcription and activation of translation by using the BiP internal ribosome entry site [29], [30]. BiP was shown to be important for HCMV virion assembly [29]. Moreover, since BiP binds to the ER stress sensors PERK, ATF6, and IRE1 and keeps them inactive, it has been suggested that BiP upregulation might also dampen the UPR [29]. We and others have also observed BiP upregulation in MCMV-infected cells (Fig. S5 and [32]), and this effect might in fact be responsible for the moderate inhibition of *Xbp1* splicing and *ERdj4* transcription observed in MCMVΔM50-infected cells (Fig. 6B and C). It should also be noted that an interaction between UL50 and BiP has been described in a previous study [50]. It remains to be investigated whether UL50 interacts with BiP directly or rather indirectly via IRE1. Collectively, the data of the present and previous studies suggest that M50/UL50 and increased BiP levels have a synergistic inhibitory effect on the IRE1-dependent signaling pathways.

Apart from the strong inhibition of the IRE1-XBP1 axis at late times postinfection, MCMV infection causes a modest induction of *Xbp1* mRNA splicing at very early times after infection (Fig. 1B, 2 hpi), which decreases within the following hours. The cause of this modest effect was not investigated in this study and remains unknown. It is unlikely that viral glycoprotein expression is responsible for this very early induction of *Xbp1* mRNA splicing as viral glycoproteins are not expressed in large quantities so early after infection. However, it is possible that the high-MOI infection itself causes ER stress, for instance, by inducing a rapid and transient Ca^2+^ release from the ER as it has been described for HSV-1 infection [51]. It also remains unknown whether the initial ER stress induction occurs only transiently, or whether it is actively inhibited by a virally induced mechanism. M50 is expressed only at late times and becomes detectable around 16 hpi (Fig. 3B). By contrast, BiP upregulation starts already 8 to 12 hpi (Fig. S5) and might contribute to inhibition of the very early *Xbp1* splicing.

By downregulating IRE1 the CMVs can avoid cellular responses that are likely detrimental for viral replication. Many XBP1s target genes encode ERAD proteins, which reduce the protein load in the ER by enhancing ER-associated protein degradation [1]. Particularly in the late phase of the viral replication cycle, when large quantities of viral glycoproteins are needed for progeny production, this counter-regulatory mechanism should have a negative impact on viral replication. XBP1s has also been reported to enhance interferon β production in dendritic cells [52], providing another good reason for the virus to block the IRE1-XBP1 pathway. Moreover, IRE1 has a role in several other pathways: Besides *Xbp1* mRNA splicing, its endoribonuclease activity also mediates cleavage and inactivation of glycoprotein-encoding mRNAs [53] as well as certain microRNAs [54] (Fig. 8C). In addition, IRE1 can initiate ER stress-induced programmed cell death by recruiting the adaptor protein TRAF2 and activating caspase-12 or JNK [16], [55], [56] (Fig. 8D). Activated JNK phosphorylates and inhibits the antiapoptotic Bcl-2 and activates proapoptotic BH3 proteins [57], [58]. One can assume that the viral mediated downregulation of IRE1, which we described in this study, inhibits all IRE1-dependent pathways. However, further in-depth studies will be necessary to fully characterize all consequences of IRE1 downregulation by M50/UL50 and a potential synergism with UL38, an HCMV protein that inhibits ER stress-induced JNK activation and apoptosis [31].

While it is clear that M50 interacts with IRE1 and downregulates IRE1 levels by reducing its half-life, the exact mechanism of IRE1 removal remains to be determined. As viruses often abuse host mechanisms for their own benefit, it is possible that the CMVs activate a cellular IRE1-inhibiting mechanism. For instance, the cellular BAX inhibtor-1 (BI-1) protein interacts with IRE1 and inhibits the IRE1-XBP1 signaling pathway [59]. However, BI-1 has not been reported to downregulate IRE1 protein levels, indicating that M50 and UL50 operate in a different manner. By contrast, the cellular protein synoviolin interacts with IRE1 and induces its ubiquitylation and degradation by the proteasome [60]. It remains to be determined whether or not M50 and UL50 operate in a similar fashion. However, the viral mediated IRE1 downregulation appears to be stronger than the one reported for synoviolin, and the preserved IRE1 downregulation by M50 in the presence of proteasome inhibitors and in ubiquitylation-deficient cells (Fig. S2) argue for a proteasome-independent mechanism.

Besides its effect on IRE1, M50 has an essential role in the export of viral capsids through the nuclear envelope. It interacts with the nuclear-localized M53 protein and facilitates primary envelopment at the inner nuclear membrane [34]. It should be worthwhile to separate the functions of M50 in capsid export and IRE1 inhibition in order to study them separately during viral infection. This is probably a very challenging task as both functions require the conserved N-terminal domain. However, with a suitable mutant virus one could investigate the importance of IRE1 inhibition for CMV replication in cell culture as well as in the mouse model.

The essential function of M50 in nuclear egress is highly conserved not only among the CMVs, but among all herpesviruses analyzed thus far [35], [36]. Hence it would be interesting to investigate whether the IRE1-downregulating function of M50 and UL50 is also conserved beyond the betaherpesviruses. Clearly, increasing evidence argues for additional, nuclear egress-unrelated functions of the M50 homologs in both alpha- and betaherpesviruses [40], [61].

## Materials and Methods

### Cells and viruses

NIH-3T3 (ATCC CRL-1658), 10.1 [62], 293T (ATCC CRL-11268); 293A (Invitrogen), telomerase-immortalized human foreskin fibroblasts (HFF) [63], ts20 cells [47], and MRC-5 (ATCC CCL-171) cells were grown under standard conditions in Dulbecco's modified Eagle's medium supplemented with 5% neonatal or 10% fetal calf serum, 100 units/ml penicillin, and 100 µg/ml streptomycin.

Wildtype MCMV, MCMV-GFP [64], MCMV-M50HA [40], and MCMV-HAm41 [65] were grown and titrated on 10.1 fibroblasts. HCMV AD169-GFP [66] was grown and titrated on HFF. MCMVΔM50 and the corresponding control virus were propagated and titrated on M50-complementing cells as described [49]. Viral titers were determined using the median tissue culture infective dose (TCID_50_) method [67].

### Plasmids and reagents

Plasmids pcDNA-hIRE1α and pCMVTAG-NEMO were purchased from Addgene, pCR3-IgM53 [34] was provided by Walter Muranyi. For pcDNA-IRE1-TEV-HA, the murine IRE1α cDNA was PCR-amplified (introducing the TEV-HA sequence with the reverse primer) and inserted between the EcoRI and XbaI sites of pcDNA3 (Invitrogen). The IRE1-TEV-HA sequence was also cloned in pBRep, an episomal replicating plasmid vector [68]. Plasmids pcDNA-hIRE1-HA, pcDNA-M50, pcDNA-M50-Flag, pcDNA-m144-Flag, and pcDNA-UL56-Flag were generated by PCR amplification and insertion of the coding sequence between the HindIII and XhoI sites of pcDNA3. Plasmids encoding N- and C-terminal truncations of M50 were generated in the same way. Deletions within the M50 variable region were made as described [39]. Substitutions of the M50 TM domain were made using a three-step PCR-based procedure essentially as described elsewhere [69]. pcDNA-UL50-Flag and pcDNA-CNX-Flag were also generated by PCR cloning using the EcoRI and XhoI sites of pcDNA3. The K599A mutation was introduced by QuikChange site-directed mutagenesis (Stratagene) into pcDNA-IRE1-HA. Transient transfections were done using ployethyleneimine (Sigma) or PolyFect transfection reagent (Qiagen) according to the manufacturer's protocol. Within each experiment, the total amount of transfected DNA was kept constant by addition of empty vector plasmid. Tunicamycin, thapsigargin, puromycin, and protease inhibitor cocktail (104 mM AEBSF, 80 µM Aprotinin, 4 mM Bestatin, 1.4 mM E-64, 2 mM Leupeptin, 1.5 mM Pepstatin A) were purchased from Sigma, MG132 from Merck, and lactacystin from Biomol.

### Retroviral transduction

HA-tagged murine IRE1α was PCR amplified, digested with BglII and XbaI, and inserted into pMSCVpuro (Clontech). Murine IRE1α with a 3xmyc tag was PCR amplified, digested with BglII and HpaI, and inserted into pMSCVhyg (Clontech). HA-tagged human IRE1α was excised from pcDNA-hIRE1-HA and inserted between the PmlI and XhoI sites of pRetroEBNA [70]. Retrovirus production using the Phoenix packaging cell line and transduction of target cells was done as described [71]. Cells transduced with MSCVpuro vectors were selected with 6 µg/ml puromycin (Sigma) and cells transduced with MSCVhyg vectors were selected with 200 µg/ml hygromycin B (PAA Laboratories).

### Affinity purification and mass spectrometry

NIH-3T3 cells were transfected with pBRep-IRE1-TEV-HA and selected as bulk culture for 14 days with 200 µg/ml hygromycin B. IRE1-TEV-HA expression was verified by immunoblot. 8×10^7^ cells were mock treated or MCMV infected at an MOI of 1. After 48 h, cells were lysed with RIPA buffer (50 mM Tris pH 7.2, 150 mM NaCl, 1% TritonX100, 0.1% SDS, 1% sodium deoxycholate, and Complete protease inhibitor cocktail [Roche]) and centrifuged for 10 min at 16000 g. Supernatants were loaded onto anti-HA 3F10 affinity columns (Roche) and washed with 20 mM Tris-HCl pH 7.5, 0.1 M NaCl, 0.1 M EDTA, 0.05% Tween-20. IRE1 and associated proteins were eluted by digestion with 100 units of AcTEV protease (Invitrogen) for 1 h at room temperature. Eluted proteins were concentrated with StrataClean resin beads, separated by SDS-PAGE, and silver-stained [52]. In-gel digestion of excised gel bands was done as described [72]. Peptide extracts were analyzed on an Orbitrap XL mass spectrometer (Thermo Scientific), online coupled to a bioinert Ultimate 3000 nano HPLCs (Thermo Scientific). Peptides were pre-concentrated on a self-packed Synergi HydroRP trapping column (100 µm ID, 4 µm particle size, 100 Å pore size, 2 cm length) and separated on a self-packed Synergi HydroRP main column (75 µm ID, 2.5 µm particle size, 100 Å pore size, 30 cm length) at 60°C and a flow rate of 270 nL/min using a binary gradient (A: 0.1% formic acid, B: 0.1% formic acid, 84% acetonitrile) ranging from 5% to 50% B in 40 min. After each sample a dedicated wash blank was applied to clean the columns. MS survey scans were acquired from 350–2000 m/z in the Orbitrap with a resolution of 60,000 using the polysiloxane m/z 445.120030 as lock [73]. The five most intense signals were subjected to MS/MS in the LTQ with a normalized collision energy of 35 and a dynamic exclusion of 30 s. Automatic gain control target values were set to 10^6^ for MS and 10^4^ for MS/MS scans. Raw data were searched with the Proteome Discoverer Software 1.2 (Thermo Scientific) and Mascot 2.2 (Matrix Science) against Uniprot mouse and murid herpesvirus 1 databases. Search settings were as follows: (i) Trypsin as enzyme with a maximum of two missed cleavage sites, (ii) carbamidomethylation of Cys as fixed modification, (iii) phosphorylation of Ser/Thr/Tyr, and oxidation of Met as variable modifications, (iv) MS and MS/MS tolerances of 10 ppm and 0.5 Da, respectively. Only proteins with at least two peptides having (i) a Mascot score above 35 and (ii) a mass deviation ≤4 ppm and (iii) between 6 and 22 amino acids, were considered for data evaluation

### Immunoprecipitation and immunoblot analysis

For immunoprecipitation 293A cells were transfected in 10 cm dishes and lysed after 24 h with RIPA buffer. Insoluble material was removed by centrifugation. Proteins were precipitated using antibodies against HA, Flag, or myc epitopes and protein A or protein G Sepharose (GE Healthcare), respectively, washed 6 times, eluted by boiling in sample buffer, and subjected to SDS-PAGE and immunoblotting.

For immunoblot analysis whole cell lysates were analyzed using antibodies against Flag epitope (M2 or F7425, Sigma), HA epitope (16B12, Covance Inc., or 3F10, Roche), myc epitope (4A6, Millipore), β actin (AC-74, Sigma), MCMV IE1 (CROMA101; provided by Stipan Jonjic, University of Rijeka, Croatia), HCMV IE1/2 (3H4; provided by Thomas Shenk, Princeton University, USA), M50 [34], M55/gB (SN1.07, provided by Stipan Jonjic), BiP (E-4, Santa Cruz); IRE1α (14C10, Cell Signaling), IRE1α[pSer724] (Novus Biologicals), XBP1s (M-186, Santa Cruz), HP1α (Cell Signaling), p53 (FL-393, Santa Cruz). Secondary antibodies coupled to horseradish peroxidase were purchased from Dako.

### Immunofluorescence

NIH-3T3 or 10.1 cells were transfected or infected on coverslips, washed with PBS, and fixed for 20 min in 4% paraformaldehyde in PBS. Cells were incubated with 50 mM ammonium chloride, permeabilized with 0.3% TritonX-100, and blocked with 0.2% cold-water fish skin gelatin (Sigma) and 2% horse serum (when the anti-M50 antiserum was used). Cells were then incubated with primary antibodies for 1 h at room temperature (RT), washed three times with PBS, and incubated for 1 h with secondary antibodies coupled to AlexaFluor555 or AlexaFluor488 (Invitrogen). Nuclei were stained using Draq5 (BioStatus). Samples were washed, mounted on slides with Aqua-Poly/Mount (Polysciences), and analyzed by confocal laser scanning microscopy using a Zeiss LSM510 Meta microscope. The Pearson correlation coefficient was calculated using JACoP for ImageJ [74].

### Pulse-chase experiment

293T cells were transfected with pcDNA-IRE1-HA and pcDNA-M50 at a 1∶4 ratio using polyethyleneimine. 48 h after transfection cells were incubated with methionine-deficient DMEM for 45 min and pulse-labeled with ^35^S-methionine (IsoLabel L-[^35^S], Izotop, Hungary) for 30 min. Cell were chased for up to 4 h in DMEM containing 50-fold excess of cold methionine. Cells were then harvested and lysed as described previously [75]. HA-tagged IRE1 was immunoprecipitated with 12CA5 anti-HA monoclonal antibody and protein G-conjugated sepharose (Santa Cruz). Immunoprecipitates were washed extensively with NET buffer containing 0.1% SDS, boiled in Laemmli sample buffer and separated by SDS-PAGE. The gel was processed for autoradiography as previously described [75].

### RNA isolation and RT-PCR

Total RNA was isolated from murine fibroblasts using innuPREP RNA Mini Kit (analytik-jena). After DNase treatment (Turbo DNA-free Kit, Ambion) cDNA was synthesized from 1 µg RNA using 200 U RevertAid H Minus Reverse Transcriptase, 100 pmol Oligo[dT]_18_, and 20 U RNase inhibitor (Thermo Scientific). For semiquantitative analysis, murine *Xbp1* was amplified by using primers 5′-AAACAGAGTAGCAGCGCAGACTGC-3′ and 5′-AAACAGAGTAGCAGCGCAGACTG C-3′. Primers 5′-GCCAGAGGAGGAACGAGCT-3′ and 5′-GGGCCTTTTCATTGTT TTCCA-3′ were used to amplify c-myc. PCR reaction was performed under the following conditions: 40 cycles of 30 s at 95°C, 30 s at 48°C, and 30 s at 72°C. PCR products were digested with PstI and analyzed on an ethidium bromide-stained agarose gel as described [76].

Quantitative RT-PCR reactions employing SYBR Green fluorescent reagent (Applied Biosystems) were run in an Applied Biosystems 7900HT Fast Real-Time PCR System. The following primers were used: 5′-GAGTCCGCAGCAGGTG-3′ and 5′-GTGTCAGAGTCCATGGGA-3′ murine *Xbp1s*, 5′-GTGTCAGAGTCCATGGGA-3′ and 5′-GTGTCAGAGTCCATGGGA-3′ for murine X*bp1u*, 5′-GAGTCCGCAGCAGGTG-3′ and 5′-CAATACCGCCAGAATCCA-3′ for human *XBP1s*, 5′-CACTCAGACTATGTGCACCTC-3′ and 5′-CAATACCGCCAGAATCCA-3′ for human *XBP1u*, 5′-ATAAAAGCCCTGATGCTGAAGC-3′ and 5′-GCCATTGGTAAAAGCACTGTGT-3′ for murine *ERdj4*, 5′-CGGCCTTTGCTGATAGTCTC-3′ and 5′-AGTTACCACCAGTCCATCGC-3′ for murine *Ire1* and 5′-CCCACTCTTCCACCTTCGATG-3′ and 5′-GTCCACCACCCTGTTGCTGTAG-3′ for human and murine *GAPDH*. Reactions were performed under the following conditions: 45 cycles of 3 s at 95°C and 30 s at 60°C. Three or four replicates were analyzed for each condition, and the relative amounts of mRNAs were calculated from the comparative threshold cycle (Ct) values by using *GAPDH* as reference.

### Accession numbers

GenBank accession numbers of proteins and genes mentioned in this study: murine ATF4 (NP_033846), ATF6 (NP_001074773), BiP (P20029), BI-1 (NP_001164507), CNX (P35564), ERdj4 (NM_013760), GAPDH (NM_008084), IRE1 (AF071777), IRE2 (Q9Z2E3), PERK (NP_034251), SYVN1 (NP_001158181), XBP1s (NM_001271730), XBP1u (NM_013842); human EDEM (NP_055489), ERdj4 (NM_012328), GAPDH (NM_002046), IRE1 (NM_001433), XBP1s (NM_001079539), XBP1u (NM_005080); MCMV IE1 (P11210), m41 (ADD10423), M50 (ADD10432), M53 (ADD10435), M55 (ADD10436), M85 (ADD10456), m144 (ADD10510); HCMV IE1 (P13202), IE2 (P19893), UL50 (P16791); HSV-1 UL56 (AEQ77088).

## Supporting Information

Figure S1 **IRE1 downregulation by M50 in the presence of M53.** NIH-3T3 cells were cotransfected with plasmids encoding IRE1-HA (1 µg) and M50 (2 µg), and Ig-tagged M53 (1 or 2 µg). After 24 h, cell lysates were analyzed by immunoblot using protein- or tag-specific antibodies.
(TIF)

Figure S2 **Downregulation of IRE1 in ts20 cells and NIH-3T3 cells treated with different lysosomal inhibitors.** (A) ts20 cells were cotransfected with plasmids encoding HA-tagged IRE1 (1 µg) and M50 (0, 2, 3 µg) and incubated at 35 or 40°C for 24 h. At 40°C, the cellular E1 ubiquitin-activating enzyme is inactive. IRE1, M50, and β-actin were detected with protein- or tag-specific antibodies. (B) NIH-3T3 cells were cotransfected with expression plasmids for IRE1-HA and M50. Transfected cells were treated for 6 h with MG132 (10 µM) or for 24 h with lactacystin (10 µM) harvested 24 h after transfection. IRE1, M50, p53 and β-actin were detected with protein- or tag-specific antibodies. Inhibition of p53 degradation by MG132 and lactacystin was used as positive control. (C) NIH-3T3 cells were cotransfected with expression plasmids for IRE1-HA and M50. 7 h after transfection, cells were treated for 24 h with a lysosomal protease-inhibitor (PI) mix (1∶200) or NH_4_Cl (10 mM). IRE1, M50, and β-actin were detected with protein- or tag-specific antibodies.
(TIF)

Figure S3 **IRE1 interaction and intracellular localization of mutant M50 proteins.** (A) 293A cells were cotransfected with expression plasmids for IRE1-HA and full-length (fl.) or mutant M50. IRE1 was immunoprecipitated (IP) with an anti-HA antibody, and coprecipitating M50 proteins were detected by immunoblot using an M50-specific antibody. The M50 1–178, 277–317 mutant was detected using an anti-Flag antibody. The same proteins were detected in whole cell lysates (WCL). LC, antibody light chain. (B) NIH-3T3 cells were cotransfected with expression plasmids for IRE1-HA and fl. or mutant M50. 24 h post transfection, cells were fixed and subjected to immunofluorescence staining using HA- and M50-specific antibodies. Cell nuclei were stained with Draq5. The Pearson correlation coefficient (PC) was determined for transfected cells.
(TIF)

Figure S4 ***Ire1* transcript levels in MCMV-infected cells.** 10.1 fibroblasts stably expressing myc-tagged IRE1 were infected with an MCMV M50 deletion mutant (ΔM50) or the parental control virus at an MOI of 3. Cells were harvested at 0, 24, and 48 hpi. *Ire1* transcripts were quantified by real-time RT-PCR. Mean ±SEM of three replicates are shown relative to uninfected cells.
(TIF)

Figure S5 **Induction of BiP during MCMV infection.** 10.1 fibroblasts were infected with MCMV at an MOI of 5 or treated with 1 µM thapsigargin (Tg) for 8 h. Cell lysates were harvested at the indicated time points, and BiP, IE1 and β-actin were detected with specific antibodies.
(TIF)

## References

[1] Smith MH, Ploegh HL, Weissman JS (2011) Road to ruin: targeting proteins for degradation in the endoplasmic reticulum. Science 334: 1086–1090. 22116878 10.1126/science.1209235 PMC3864754

[2] Zhang L, Wang A (2012) Virus-induced ER stress and the unfolded protein response. Front Plant Sci 3: 293. 23293645 10.3389/fpls.2012.00293 PMC3531707

[3] Tirosh B, Iwakoshi NN, Lilley BN, Lee AH, Glimcher LH, et al (2005) Human cytomegalovirus protein US11 provokes an unfolded protein response that may facilitate the degradation of class I major histocompatibility complex products. J Virol 79: 2768–2779. 15708995 10.1128/JVI.79.5.2768-2779.2005 PMC548438

[4] Harding HP, Calfon M, Urano F, Novoa I, Ron D (2002) Transcriptional and translational control in the Mammalian unfolded protein response. Annu Rev Cell Dev Biol 18: 575–599. 12142265 10.1146/annurev.cellbio.18.011402.160624

[5] Zhang K, Kaufman RJ (2004) Signaling the unfolded protein response from the endoplasmic reticulum. J Biol Chem 279: 25935–25938. 15070890 10.1074/jbc.R400008200

[6] Hamanaka RB, Bennett BS, Cullinan SB, Diehl JA (2005) PERK and GCN2 contribute to eIF2alpha phosphorylation and cell cycle arrest after activation of the unfolded protein response pathway. Mol Biol Cell 16: 5493–5501. 16176978 10.1091/mbc.E05-03-0268 PMC1289396

[7] Yang W, Hinnebusch AG (1996) Identification of a regulatory subcomplex in the guanine nucleotide exchange factor eIF2B that mediates inhibition by phosphorylated eIF2. Mol Cell Biol 16: 6603–6616. 8887689 10.1128/mcb.16.11.6603 PMC231662

[8] McCullough KD, Martindale JL, Klotz LO, Aw TY, Holbrook NJ (2001) Gadd153 sensitizes cells to endoplasmic reticulum stress by down-regulating Bcl2 and perturbing the cellular redox state. Mol Cell Biol 21: 1249–1259. 11158311 10.1128/MCB.21.4.1249-1259.2001 PMC99578

[9] Marciniak SJ, Yun CY, Oyadomari S, Novoa I, Zhang Y, et al (2004) CHOP induces death by promoting protein synthesis and oxidation in the stressed endoplasmic reticulum. Genes Dev 18: 3066–3077. 15601821 10.1101/gad.1250704 PMC535917

[10] Ye J, Rawson RB, Komuro R, Chen X, Dave UP, et al (2000) ER stress induces cleavage of membrane-bound ATF6 by the same proteases that process SREBPs. Mol Cell 6: 1355–1364. 11163209 10.1016/s1097-2765(00)00133-7

[11] Thuerauf DJ, Marcinko M, Belmont PJ, Glembotski CC (2007) Effects of the isoform-specific characteristics of ATF6 alpha and ATF6 beta on endoplasmic reticulum stress response gene expression and cell viability. J Biol Chem 282: 22865–22878. 17522056 10.1074/jbc.M701213200

[12] Hetz C, Martinon F, Rodriguez D, Glimcher LH (2011) The Unfolded Protein Response: Integrating Stress Signals Through the Stress Sensor IRE1α. Physiol Rev 91: 1219–1243. 22013210 10.1152/physrev.00001.2011

[13] Wang XZ, Harding HP, Zhang Y, Jolicoeur EM, Kuroda M, et al (1998) Cloning of mammalian Ire1 reveals diversity in the ER stress responses. EMBO J 17: 5708–5717. 9755171 10.1093/emboj/17.19.5708 PMC1170899

[14] Lee K, Tirasophon W, Shen X, Michalak M, Prywes R, et al (2002) IRE1-mediated unconventional mRNA splicing and S2P-mediated ATF6 cleavage merge to regulate XBP1 in signaling the unfolded protein response. Genes Dev 16: 452–466. 11850408 10.1101/gad.964702 PMC155339

[15] Calfon M, Zeng H, Urano F, Till JH, Hubbard SR, et al (2002) IRE1 couples endoplasmic reticulum load to secretory capacity by processing the XBP-1 mRNA. Nature 415: 92–96. 11780124 10.1038/415092a

[16] Szegezdi E, Logue SE, Gorman AM, Samali A (2006) Mediators of endoplasmic reticulum stress-induced apoptosis. EMBO Rep 7: 880–885. 16953201 10.1038/sj.embor.7400779 PMC1559676

[17] Tardif KD, Mori K, Kaufman RJ, Siddiqui A (2004) Hepatitis C virus suppresses the IRE1-XBP1 pathway of the unfolded protein response. J Biol Chem 279: 17158–17164. 14960590 10.1074/jbc.M312144200

[18] Jordan R, Wang L, Graczyk TM, Block TM, Romano PR (2002) Replication of a cytopathic strain of bovine viral diarrhea virus activates PERK and induces endoplasmic reticulum stress-mediated apoptosis of MDBK cells. J Virol 76: 9588–9599. 12208938 10.1128/JVI.76.19.9588-9599.2002 PMC136515

[19] Zheng Y, Gao B, Ye L, Kong L, Jing W, et al (2005) Hepatitis C virus non-structural protein NS4B can modulate an unfolded protein response. J Microbiol 43: 529–536. 16410770

[20] Tardif KD, Waris G, Siddiqui A (2005) Hepatitis C virus, ER stress, and oxidative stress. Trends Microbiol 13: 159–163. 15817385 10.1016/j.tim.2005.02.004

[21] Lee DY, Lee J, Sugden B (2009) The unfolded protein response and autophagy: herpesviruses rule!. J Virol 83: 1168–1172. 18787009 10.1128/JVI.01358-08 PMC2620921

[22] Mulvey M, Arias C, Mohr I (2007) Maintenance of endoplasmic reticulum (ER) homeostasis in herpes simplex virus type 1-infected cells through the association of a viral glycoprotein with PERK, a cellular ER stress sensor. J Virol 81: 3377–3390. 17229688 10.1128/JVI.02191-06 PMC1866074

[23] Carpenter JE, Jackson W, Benetti L, Grose C (2011) Autophagosome formation during varicella-zoster virus infection following endoplasmic reticulum stress and the unfolded protein response. J Virol 85: 9414–9424. 21752906 10.1128/JVI.00281-11 PMC3165774

[24] Lee DY, Sugden B (2008) The LMP1 oncogene of EBV activates PERK and the unfolded protein response to drive its own synthesis. Blood 111: 2280–2289. 18042799 10.1182/blood-2007-07-100032 PMC2234060

[25] Bhende PM, Dickerson SJ, Sun X, Feng WH, Kenney SC (2007) X-box-binding protein 1 activates lytic Epstein-Barr virus gene expression in combination with protein kinase D. J Virol. 81: 7363–7370. 10.1128/JVI.00154-07 PMC1933364 17494074

[26] Mocarski ES, Shenk T, Pass RF (2007) Cytomegaloviruses. In: Knipe DM, Howley PM, editors. Fields Virology 5th edn. Philadelphia: Lippincott, Williams and Wilkins. pp. 2701–2772.

[27] Isler JA, Skalet AH, Alwine JC (2005) Human cytomegalovirus infection activates and regulates the unfolded protein response. J Virol 79: 6890–6899. 15890928 10.1128/JVI.79.11.6890-6899.2005 PMC1112127

[28] Yu Y, Pierciey FJ Jr, Maguire TG, Alwine JC (2013) PKR-Like Endoplasmic Reticulum Kinase Is Necessary for Lipogenic Activation during HCMV Infection. PLoS Pathog 9: e1003266. 23592989 10.1371/journal.ppat.1003266 PMC3617203

[29] Buchkovich NJ, Maguire TG, Yu Y, Paton AW, Paton JC, et al (2008) Human cytomegalovirus specifically controls the levels of the endoplasmic reticulum chaperone BiP/GRP78, which is required for virion assembly. J Virol 82: 31–39. 17942541 10.1128/JVI.01881-07 PMC2224369

[30] Buchkovich NJ, Yu Y, Pierciey FJ Jr, Alwine JC (2010) Human cytomegalovirus induces the endoplasmic reticulum chaperone BiP through increased transcription and activation of translation by using the BiP internal ribosome entry site. J Virol 84: 11479–11486. 20739513 10.1128/JVI.01330-10 PMC2953172

[31] Xuan B, Qian Z, Torigoi E, Yu D (2009) Human cytomegalovirus protein pUL38 induces ATF4 expression, inhibits persistent JNK phosphorylation, and suppresses endoplasmic reticulum stress-induced cell death. J Virol 83: 3463–3474. 19193809 10.1128/JVI.02307-08 PMC2663240

[32] Qian Z, Xuan B, Chapa TJ, Gualberto N, Yu D (2012) Murine cytomegalovirus targets transcription factor ATF4 to exploit the unfolded protein response. J Virol 86: 6712–6723. 22496230 10.1128/JVI.00200-12 PMC3393534

[33] Korennykh A, Walter P (2012) Structural basis of the unfolded protein response. Annu Rev Cell Dev Biol 28: 251–277. 23057742 10.1146/annurev-cellbio-101011-155826

[34] Muranyi W, Haas J, Wagner M, Krohne G, Koszinowski UH (2002) Cytomegalovirus recruitment of cellular kinases to dissolve the nuclear lamina. Science 297: 854–857. 12161659 10.1126/science.1071506

[35] Johnson DC, Baines JD (2011) Herpesviruses remodel host membranes for virus egress. Nat Rev Microbiol 9: 382–394. 21494278 10.1038/nrmicro2559

[36] Mettenleiter TC, Muller F, Granzow H, Klupp BG (2013) The way out: what we know and do not know about herpesvirus nuclear egress. Cell Microbiol 15: 170–178. 23057731 10.1111/cmi.12044

[37] Lai CW, Otero JH, Hendershot LM, Snapp E (2012) ERdj4 protein is a soluble endoplasmic reticulum (ER) DnaJ family protein that interacts with ER-associated degradation machinery. J Biol Chem 287: 7969–7978. 22267725 10.1074/jbc.M111.311290 PMC3318715

[38] Bubeck A, Wagner M, Ruzsics Z, Lotzerich M, Iglesias M, et al (2004) Comprehensive mutational analysis of a herpesvirus gene in the viral genome context reveals a region essential for virus replication. J Virol 78: 8026–8035. 15254174 10.1128/JVI.78.15.8026-8035.2004 PMC446129

[39] Rupp B, Ruzsics Z, Buser C, Adler B, Walther P, et al (2007) Random screening for dominant-negative mutants of the cytomegalovirus nuclear egress protein M50. J Virol 81: 5508–5517. 17376929 10.1128/JVI.02796-06 PMC1900260

[40] Lemnitzer F, Raschbichler V, Kolodziejczak D, Israel L, Imhof A, et al (2013) Mouse cytomegalovirus egress protein pM50 interacts with cellular endophilin-A2. Cell Microbiol 15: 335–351. 23189961 10.1111/cmi.12080

[41] Kattenhorn LM, Mills R, Wagner M, Lomsadze A, Makeev V, et al (2004) Identification of proteins associated with murine cytomegalovirus virions. J Virol 78: 11187–11197. 15452238 10.1128/JVI.78.20.11187-11197.2004 PMC521832

[42] Hetz C, Bernasconi P, Fisher J, Lee AH, Bassik MC, et al (2006) Proapoptotic BAX and BAK modulate the unfolded protein response by a direct interaction with IRE1alpha. Science 312: 572–576. 16645094 10.1126/science.1123480

[43] Rodriguez DA, Zamorano S, Lisbona F, Rojas-Rivera D, Urra H, et al (2012) BH3-only proteins are part of a regulatory network that control the sustained signalling of the unfolded protein response sensor IRE1alpha. EMBO J 31: 2322–2335. 22510886 10.1038/emboj.2012.84 PMC3364744

[44] Brune W, Nevels M, Shenk T (2003) Murine cytomegalovirus m41 open reading frame encodes a Golgi-localized antiapoptotic protein. J Virol 77: 11633–11643. 14557649 10.1128/JVI.77.21.11633-11643.2003 PMC229354

[45] Ott M, Tascher G, Hassdenteufel S, Zimmermann R, Haas J, et al (2011) Functional characterization of the essential tail-anchor of the HSV-1 nuclear egress protein UL34. J Gen Virol 92: 2734–45. 21832006 10.1099/vir.0.032730-0

[46] Tirasophon W, Lee K, Callaghan B, Welihinda A, Kaufman RJ (2000) The endoribonuclease activity of mammalian IRE1 autoregulates its mRNA and is required for the unfolded protein response. Genes Dev 14: 2725–2736. 11069889 10.1101/gad.839400 PMC317029

[47] Chowdary DR, Dermody JJ, Jha KK, Ozer HL (1994) Accumulation of p53 in a mutant cell line defective in the ubiquitin pathway. Mol Cell Biol 14: 1997–2003. 8114731 10.1128/mcb.14.3.1997-2003.1994 PMC358559

[48] Schnee M, Ruzsics Z, Bubeck A, Koszinowski UH (2006) Common and specific properties of herpesvirus UL34/UL31 protein family members revealed by protein complementation assay. J Virol 80: 11658–11666. 17005637 10.1128/JVI.01662-06 PMC1642623

[49] Mohr H, Mohr CA, Schneider MR, Scrivano L, Adler B, et al (2012) Cytomegalovirus replicon-based regulation of gene expression in vitro and in vivo. PLoS Pathog 8: e1002728. 22685399 10.1371/journal.ppat.1002728 PMC3369935

[50] Buchkovich NJ, Maguire TG, Alwine JC (2010) Role of the endoplasmic reticulum chaperone BiP, SUN domain proteins, and dynein in altering nuclear morphology during human cytomegalovirus infection. J Virol 84: 7005–7017. 20484513 10.1128/JVI.00719-10 PMC2898220

[51] Cheshenko N, Del Rosario B, Woda C, Marcellino D, Satlin LM, et al (2003) Herpes simplex virus triggers activation of calcium-signaling pathways. J Cell Biol 163: 283–293. 14568989 10.1083/jcb.200301084 PMC2173509

[52] Hu F, Yu X, Wang H, Zuo D, Guo C, et al (2011) ER stress and its regulator X-box-binding protein-1 enhance polyIC-induced innate immune response in dendritic cells. Eur J Immunol 41: 1086–1097. 21400498 10.1002/eji.201040831 PMC3157298

[53] Hollien J, Weissman JS (2006) Decay of endoplasmic reticulum-localized mRNAs during the unfolded protein response. Science 313: 104–107. 16825573 10.1126/science.1129631

[54] Upton JP, Wang L, Han D, Wang ES, Huskey NE, et al (2012) IRE1alpha Cleaves Select microRNAs during ER Stress to Derepress Translation of Proapoptotic Caspase-2. Science 338: 818–822. 23042294 10.1126/science.1226191 PMC3742121

[55] Yoneda T, Imaizumi K, Oono K, Yui D, Gomi F, et al (2001) Activation of caspase-12, an endoplastic reticulum (ER) resident caspase, through tumor necrosis factor receptor-associated factor 2-dependent mechanism in response to the ER stress. J Biol Chem 276: 13935–13940. 11278723 10.1074/jbc.M010677200

[56] Urano F, Wang X, Bertolotti A, Zhang Y, Chung P, et al (2000) Coupling of stress in the ER to activation of JNK protein kinases by transmembrane protein kinase IRE1. Science 287: 664–666. 10650002 10.1126/science.287.5453.664

[57] Lei K, Davis RJ (2003) JNK phosphorylation of Bim-related members of the Bcl2 family induces Bax-dependent apoptosis. Proc Natl Acad Sci U S A 100: 2432–2437. 12591950 10.1073/pnas.0438011100 PMC151358

[58] Putcha GV, Le S, Frank S, Besirli CG, Clark K, et al (2003) JNK-mediated BIM phosphorylation potentiates BAX-dependent apoptosis. Neuron 38: 899–914. 12818176 10.1016/s0896-6273(03)00355-6

[59] Lisbona F, Rojas-Rivera D, Thielen P, Zamorano S, Todd D, et al (2009) BAX inhibitor-1 is a negative regulator of the ER stress sensor IRE1alpha. Mol Cell 33: 679–691. 19328063 10.1016/j.molcel.2009.02.017 PMC2818874

[60] Gao B, Lee SM, Chen A, Zhang J, Zhang DD, et al (2008) Synoviolin promotes IRE1 ubiquitination and degradation in synovial fibroblasts from mice with collagen-induced arthritis. EMBO Rep 9: 480–485. 18369366 10.1038/embor.2008.37 PMC2373369

[61] Haugo AC, Szpara ML, Parsons L, Enquist LW, Roller RJ (2011) Herpes simplex virus 1 pUL34 plays a critical role in cell-to-cell spread of virus in addition to its role in virus replication. J Virol 85: 7203–7215. 21561917 10.1128/JVI.00262-11 PMC3126596

[62] Harvey DM, Levine AJ (1991) p53 alteration is a common event in the spontaneous immortalization of primary BALB/c murine embryo fibroblasts. Genes Dev 5: 2375–2385. 1752433 10.1101/gad.5.12b.2375

[63] Bresnahan WA, Hultman GE, Shenk T (2000) Replication of wild-type and mutant human cytomegalovirus in life-extended human diploid fibroblasts. J Virol 74: 10816–10818. 11044129 10.1128/jvi.74.22.10816-10818.2000 PMC110959

[64] Brune W, Menard C, Heesemann J, Koszinowski UH (2001) A ribonucleotide reductase homolog of cytomegalovirus and endothelial cell tropism. Science 291: 303–305. 11209080 10.1126/science.291.5502.303

[65] Çam M (2009) The antiapoptotic function of the murine cytomegalovirus m41 locus. [Doctoral Thesis]. Berlin: Freie Universität. 106 p.

[66] Marschall M, Freitag M, Weiler S, Sorg G, Stamminger T (2000) Recombinant green fluorescent protein-expressing human cytomegalovirus as a tool for screening antiviral agents. Antimicrob Agents Chemother 44: 1588–1597. 10817714 10.1128/aac.44.6.1588-1597.2000 PMC89918

[67] Mahy BWJ, Kangro HO (1996) Virology methods manual. San Diego, CA: Academic Press.

[68] Hobom U, Brune W, Messerle M, Hahn G, Koszinowski UH (2000) Fast screening procedures for random transposon libraries of cloned herpesvirus genomes: mutational analysis of human cytomegalovirus envelope glycoprotein genes. J Virol 74: 7720–7729. 10933677 10.1128/jvi.74.17.7720-7729.2000 PMC112300

[69] Liang L, Baines JD (2005) Identification of an essential domain in the herpes simplex virus 1 UL34 protein that is necessary and sufficient to interact with UL31 protein. J Virol 79: 3797–3806. 15731273 10.1128/JVI.79.6.3797-3806.2005 PMC1075724

[70] Kinsella TM, Nolan GP (1996) Episomal vectors rapidly and stably produce high-titer recombinant retrovirus. Hum Gene Ther 7: 1405–1413. 8844199 10.1089/hum.1996.7.12-1405

[71] Swift S, Lorens J, Achacoso P, Nolan GP (2001) Rapid production of retroviruses for efficient gene delivery to mammalian cells using 293T cell-based systems. Curr Protoc Immunol Chapter 10: Unit 10 17C. 10.1002/0471142735.im1017cs31 18432682

[72] Burkhart JM, Schumbrutzki C, Wortelkamp S, Sickmann A, Zahedi RP (2012) Systematic and quantitative comparison of digest efficiency and specificity reveals the impact of trypsin quality on MS-based proteomics. J Proteomics 75: 1454–1462. 22166745 10.1016/j.jprot.2011.11.016

[73] Olsen JV, de Godoy LM, Li G, Macek B, Mortensen P, et al (2005) Parts per million mass accuracy on an Orbitrap mass spectrometer via lock mass injection into a C-trap. Mol Cell Proteomics 4: 2010–2021. 16249172 10.1074/mcp.T500030-MCP200

[74] Bolte S, Cordelieres FP (2006) A guided tour into subcellular colocalization analysis in light microscopy. J Microsc 224: 213–232. 17210054 10.1111/j.1365-2818.2006.01706.x

[75] Goldfinger M, Laviad EL, Hadar R, Shmuel M, Dagan A, et al (2009) De novo ceramide synthesis is required for N-linked glycosylation in plasma cells. J Immunol 182: 7038–7047. 19454701 10.4049/jimmunol.0802990

[76] Han D, Upton JP, Hagen A, Callahan J, Oakes SA, et al (2008) A kinase inhibitor activates the IRE1alpha RNase to confer cytoprotection against ER stress. Biochem Biophys Res Commun 365: 777–783. 18035051 10.1016/j.bbrc.2007.11.040

