# Supplementary figures and images for: Cytomegalovirus Downregulates IRE1 to Repress the Unfolded Protein Response
Source: PLoS Pathog. 2013 Aug 8;9(8):e1003544. doi: 10.1371/journal.ppat.1003544 (PMC3738497; doi:10.1371/journal.ppat.1003544)

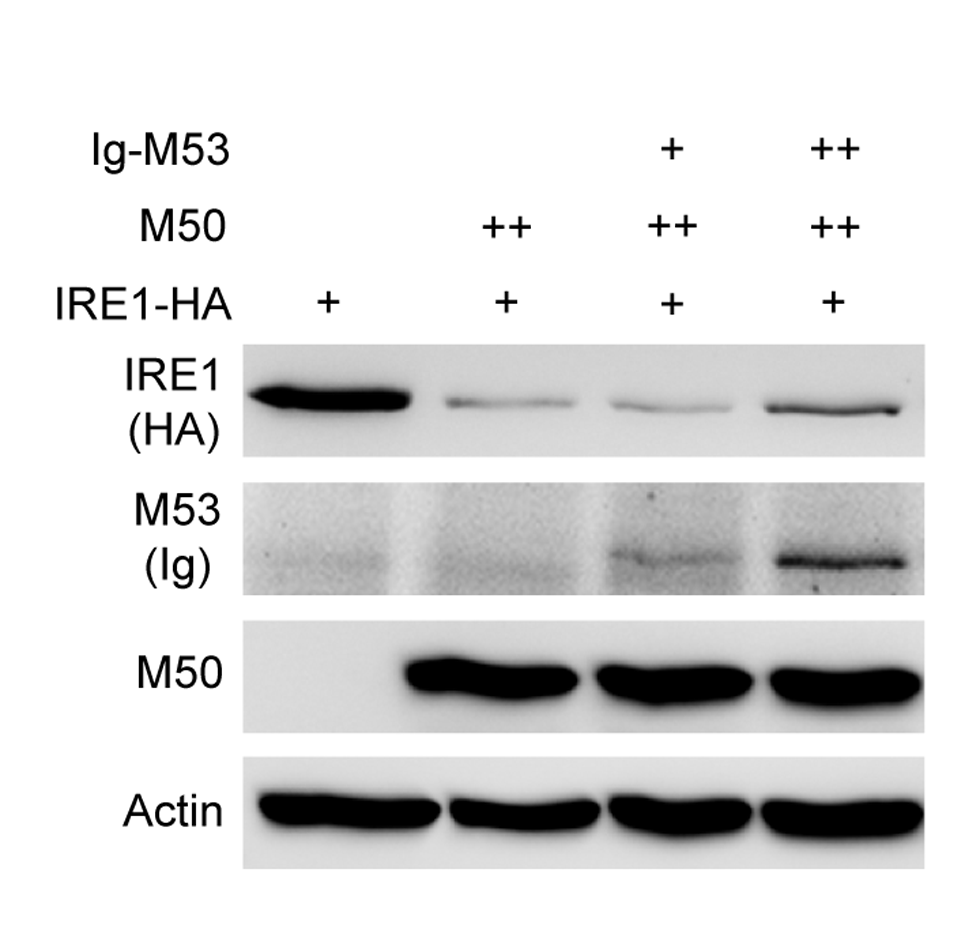

Supplement: Figure S1 — IRE1 downregulation by M50 in the presence of M53. NIH-3T3 cells were cotransfected with plasmids encoding IRE1-HA (1 µg) and M50 (2 µg), and Ig-tagged M53 (1 or 2 µg). After 24 h, cell lysates were analyzed by immunoblot using protein- or tag-specific antibodies. (TIF) [file ppat.1003544.s001.tif]

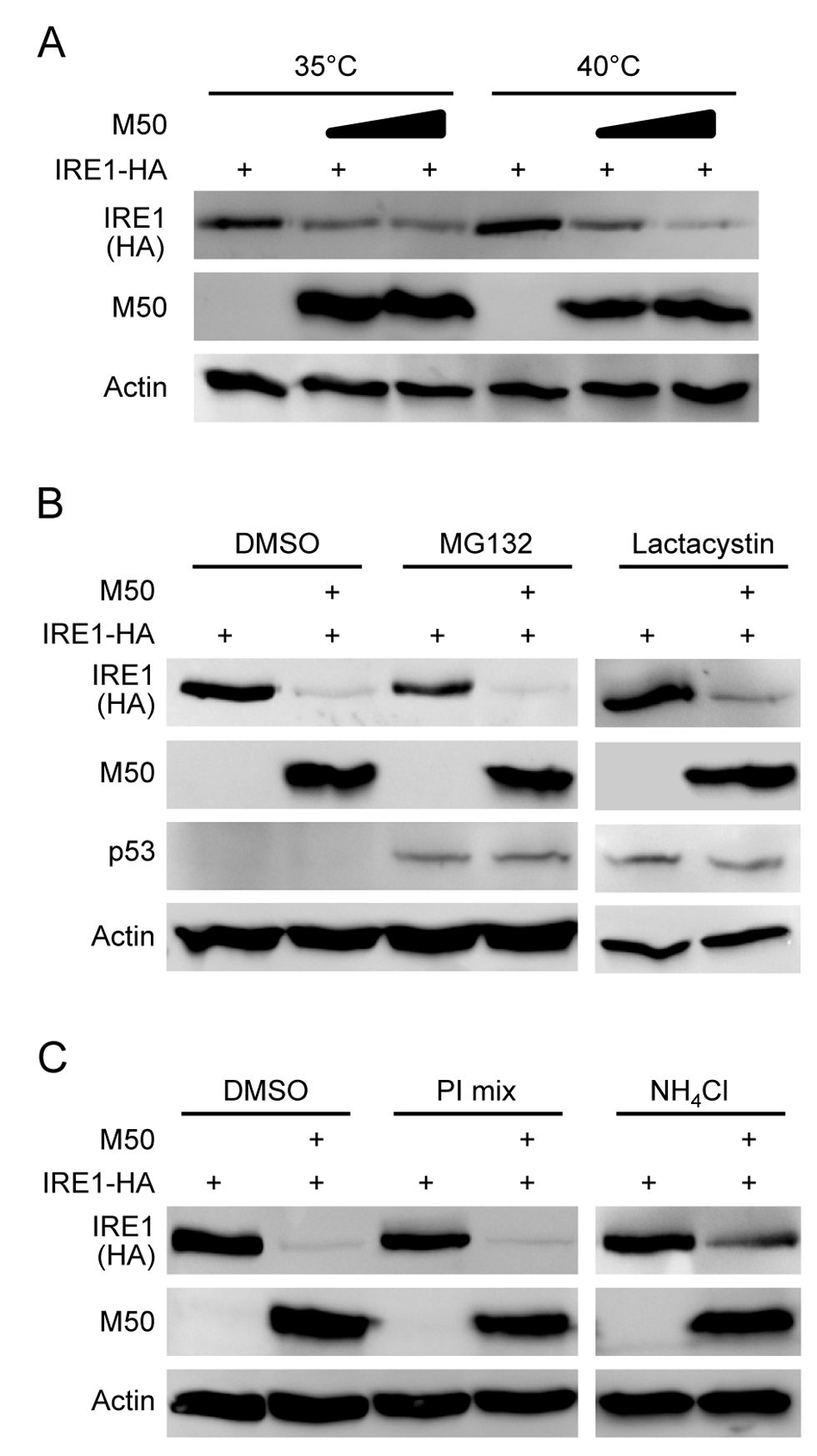

Supplement: Figure S2 — Downregulation of IRE1 in ts20 cells and NIH-3T3 cells treated with different lysosomal inhibitors. (A) ts20 cells were cotransfected with plasmids encoding HA-tagged IRE1 (1 µg) and M50 (0, 2, 3 µg) and incubated at 35 or 40°C for 24 h. At 40°C, the cellular E1 ubiquitin-activating enzyme is inactive. IRE1, M50, and β-actin were detected with protein- or tag-specific antibodies. (B) NIH-3T3 cells were cotransfected with expression plasmids for IRE1-HA and M50. Transfected cells were treated for 6 h with MG132 (10 µM) or for 24 h with lactacystin (10 µM) harvested 24 h after transfection. IRE1, M50, p53 and β-actin were detected with protein- or tag-specific antibodies. Inhibition of p53 degradation by MG132 and lactacystin was used as positive control. (C) NIH-3T3 cells were cotransfected with expression plasmids for IRE1-HA and M50. 7 h after transfection, cells were treated for 24 h with a lysosomal protease-inhibitor (PI) mix (1∶200) or NH4Cl (10 mM). IRE1, M50, and β-actin were detected with protein- or tag-specific antibodies. (TIF) [file ppat.1003544.s002.tif]

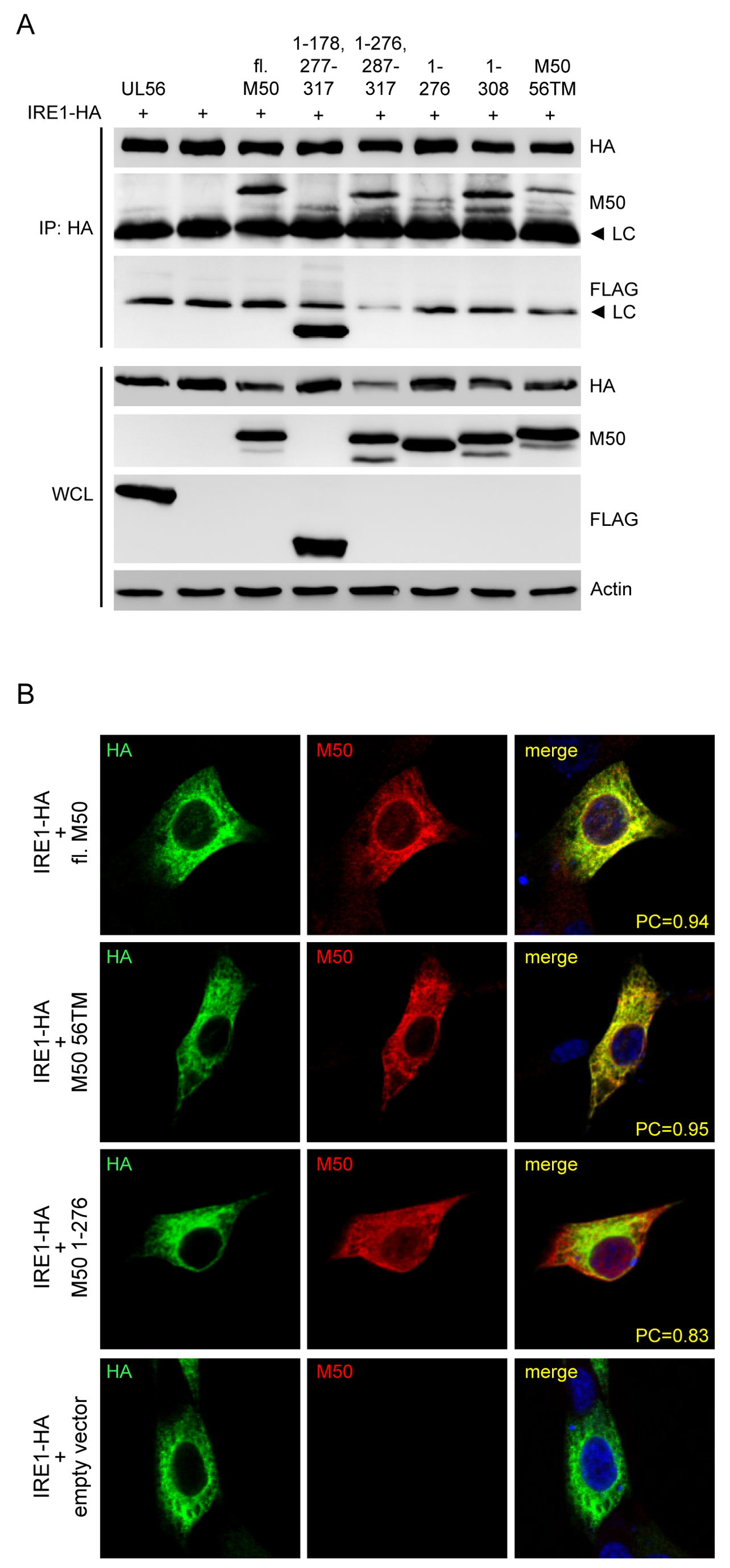

Supplement: Figure S3 — IRE1 interaction and intracellular localization of mutant M50 proteins. (A) 293A cells were cotransfected with expression plasmids for IRE1-HA and full-length (fl.) or mutant M50. IRE1 was immunoprecipitated (IP) with an anti-HA antibody, and coprecipitating M50 proteins were detected by immunoblot using an M50-specific antibody. The M50 1–178, 277–317 mutant was detected using an anti-Flag antibody. The same proteins were detected in whole cell lysates (WCL). LC, antibody light chain. (B) NIH-3T3 cells were cotransfected with expression plasmids for IRE1-HA and fl. or mutant M50. 24 h post transfection, cells were fixed and subjected to immunofluorescence staining using HA- and M50-specific antibodies. Cell nuclei were stained with Draq5. The Pearson correlation coefficient (PC) was determined for transfected cells. (TIF) [file ppat.1003544.s003.tif]

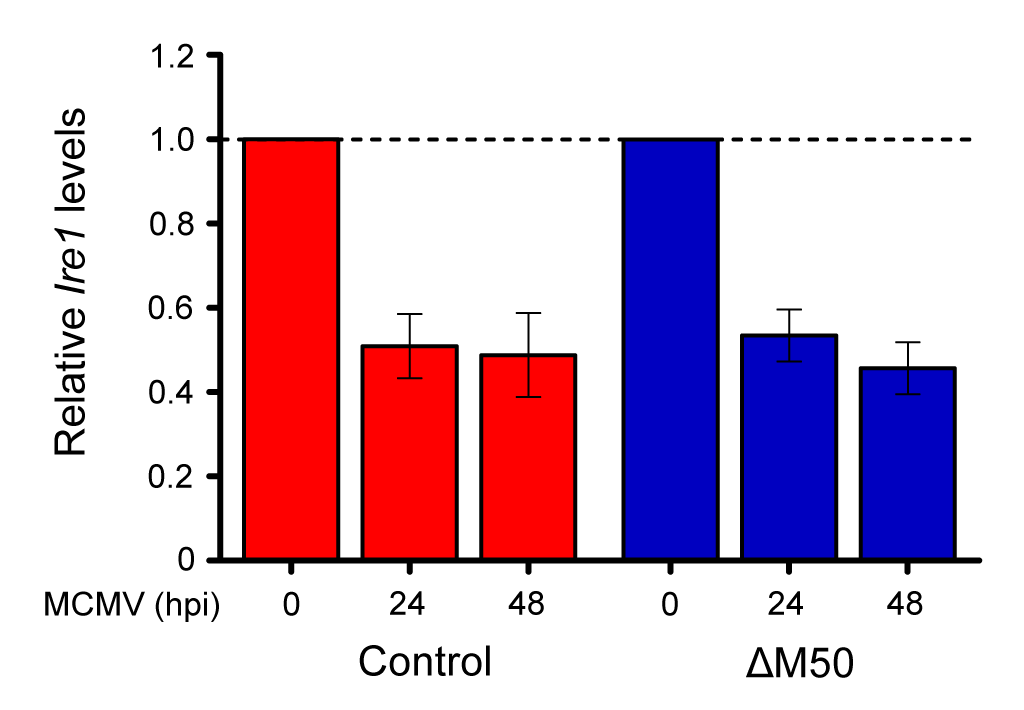

Supplement: Figure S4 — Ire1 transcript levels in MCMV-infected cells. 10.1 fibroblasts stably expressing myc-tagged IRE1 were infected with an MCMV M50 deletion mutant (ΔM50) or the parental control virus at an MOI of 3. Cells were harvested at 0, 24, and 48 hpi. Ire1 transcripts were quantified by real-time RT-PCR. Mean ±SEM of three replicates are shown relative to uninfected cells. (TIF) [file ppat.1003544.s004.tif]

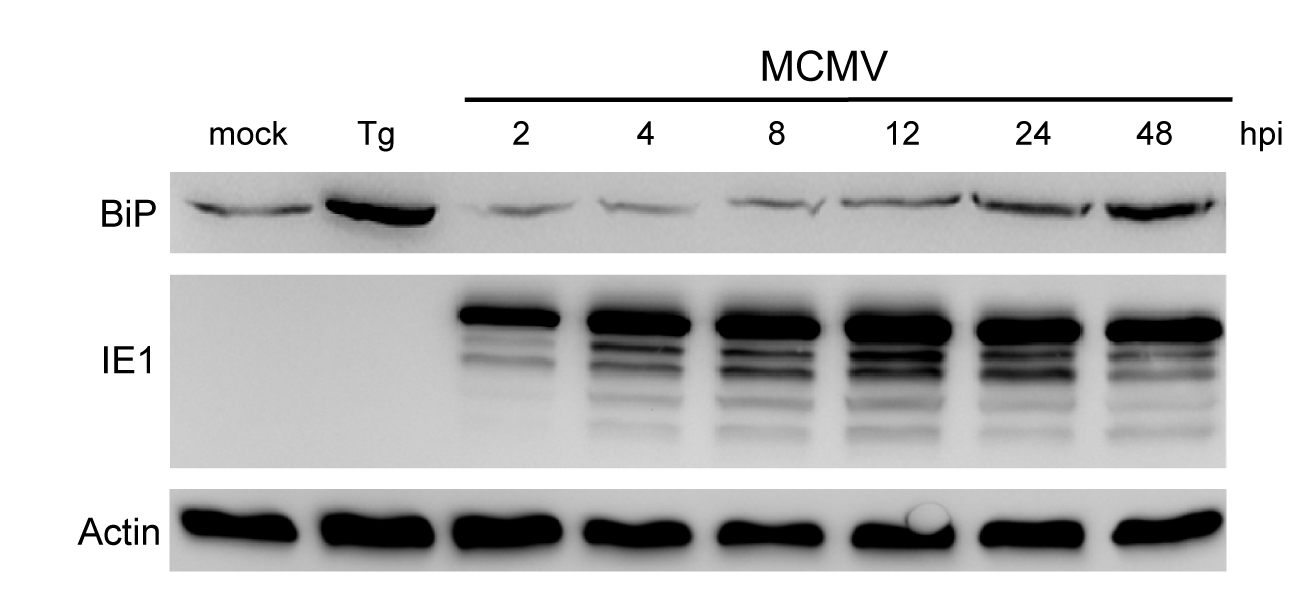

Supplement: Figure S5 — Induction of BiP during MCMV infection. 10.1 fibroblasts were infected with MCMV at an MOI of 5 or treated with 1 µM thapsigargin (Tg) for 8 h. Cell lysates were harvested at the indicated time points, and BiP, IE1 and β-actin were detected with specific antibodies. (TIF) [file ppat.1003544.s005.tif]
